# Molecular responses of agroinfiltrated *Nicotiana benthamiana* leaves expressing suppressor of silencing P19 and influenza virus‐like particles

**DOI:** 10.1111/pbi.14247

**Published:** 2023-12-02

**Authors:** Louis‐Philippe Hamel, Rachel Tardif, Francis Poirier‐Gravel, Asieh Rasoolizadeh, Chantal Brosseau, Geneviève Giroux, Jean‐François Lucier, Marie‐Claire Goulet, Adam Barrada, Marie‐Ève Paré, Élise Roussel, Marc‐André Comeau, Pierre‐Olivier Lavoie, Peter Moffett, Dominique Michaud, Marc‐André D'Aoust

**Affiliations:** ^1^ Medicago Inc. Québec Québec Canada; ^2^ Centre SÈVE, Faculté des Sciences, Département de Biologie Université de Sherbrooke Sherbrooke Québec Canada; ^3^ Centre de Recherche et d'innovation sur les Végétaux, Département de Phytologie Université Laval Québec Québec Canada

**Keywords:** Plant immunity, Influenza hemagglutinin, Virus‐like particles, Transient *Agrobacterium*‐mediated expression, *Nicotiana benthamiana*, Plant molecular farming

## Abstract

The production of influenza vaccines in plants is achieved through transient expression of viral hemagglutinins (HAs), a process mediated by the bacterial vector *Agrobacterium tumefaciens*. HA proteins are then produced and matured through the secretory pathway of plant cells, before being trafficked to the plasma membrane where they induce formation of virus‐like particles (VLPs). Production of VLPs unavoidably impacts plant cells, as do viral suppressors of RNA silencing (VSRs) that are co‐expressed to increase recombinant protein yields. However, little information is available on host molecular responses to foreign protein expression. This work provides a comprehensive overview of molecular changes occurring in *Nicotiana benthamiana* leaf cells transiently expressing the VSR P19, or co‐expressing P19 and an influenza HA. Our data identifies general responses to *Agrobacterium*‐mediated expression of foreign proteins, including shutdown of chloroplast gene expression, activation of oxidative stress responses and reinforcement of the plant cell wall through lignification. Our results also indicate that P19 expression promotes salicylic acid (SA) signalling, a process dampened by co‐expression of the HA protein. While reducing P19 level, HA expression also induces specific signatures, with effects on lipid metabolism, lipid distribution within membranes and oxylipin‐related signalling. When producing VLPs, dampening of P19 responses thus likely results from lower expression of the VSR, crosstalk between SA and oxylipin pathways, or a combination of both outcomes. Consistent with the upregulation of oxidative stress responses, we finally show that reduction of oxidative stress damage through exogenous application of ascorbic acid improves plant biomass quality during production of VLPs.

## Introduction

Hemagglutinins (HAs) are trimeric glycoproteins found on the surface of influenza viruses (a list of all abbreviations is available in the Appendix S1 section). HAs are essential for infection as they bind to sialic acid receptors located in the plasma membrane (PM) of epithelial cells from the host respiratory system (Matrosovich *et al*., 2015). Vaccination is broadly recognized as one of the most effective methods to prevent influenza infections and limit societal and economic burden associated to seasonal and pandemic strains of the virus (Ortiz de Lejarazu‐Leonardo *et al*., 2021). Host neutralizing antibodies that recognize the receptor‐binding domain of HA proteins block interaction of the virus with host cells and therefore represent a key correlate of protection against influenza. Correspondingly, HA antigens constitute the main target for commercial production of influenza vaccines. Egg‐based approaches are still the most widely employed to produce these vaccines, however methods have remained mostly unchanged since their introduction more than 70 years ago (Bouvier, 2018). Egg‐based vaccine production is also constrained by certain drawbacks, including year‐to‐year (or strain‐to‐strain) variation in effectiveness and dependency on pathogen‐free egg supplies (Rajaram *et al*., 2020), which may become an issue in case of avian influenza pandemics.

Transient expression mediated by *Rhizobium radiobacter* (commonly known and hereafter referred to as *Agrobacterium tumefaciens*) is a powerful tool to express recombinant proteins in plants (Sainsbury and Lomonossoff, 2014). *Agrobacterium*‐mediated expression (agroinfiltration) is for instance widely employed to study protein function and/or sub‐cellular localization *in planta*. When performed at large‐scale, agroinfiltration also represents a very effective ‘molecular farming’ approach, a term collectively referring to strategies in which plant cells are used as factories to produce biopharmaceutical products (Chung *et al*., 2022). As such, molecular farming offers alternative approaches to classical protein production systems, and it has become helpful in the fight against global health issues, including novel influenza virus strains and emergence of new infectious diseases.

Using a molecular farming approach in *N. benthamiana* leaf cells, the biopharmaceutical company Medicago has developed a method to produce influenza vaccines via the transient expression of recombinant influenza *HA* genes (D'Aoust *et al*., 2008; Landry *et al*., 2010). Engineered to enter the secretory pathway of plant cells more efficiently, newly synthesized HAs are trafficked to the PM, where they end up in discrete microdomains called lipid rafts. When sufficient HA proteins have accumulated, PM curvature is altered, resulting in budding of the so‐called virus‐like particles (VLPs; D'Aoust *et al*., 2008). These nanoscale assemblies comprise trimer clusters of the engineered HA protein, as well as a lipid envelope derived from the PM of plant cells. Structurally, VLPs and influenza virions are similar in size and shape, however the former lack other viral proteins such as the surface exposed neuraminidase, in addition to being devoid of the genetic components required for replication. Once purified and formulated into vaccine candidates, VLPs activate an immune response protecting newly immunized hosts from subsequent infection by the virus (Landry *et al*., 2010).

To sustain commercial production of influenza vaccines in plants, transient expression of HA proteins was optimized so high quantities of VLPs are produced. This includes co‐expression of the viral suppressor of RNA silencing (VSR) P19 (Silhavy *et al*., 2002), which prevents silencing of *HA* genes delivered by the *Agrobacterium*. Since plant cells produce high amounts of HA proteins and supply membrane lipids to support the formation of VLP envelopes, this expression system unavoidably triggers energy and resource demanding processes, including elevated transcription and translation rates, as well as plant immunity. While the effects of disarmed *Agrobacterium* strains were previously investigated (Anand *et al*., 2008; Ditt *et al*., 2006), the combined impacts of agroinfiltration and foreign protein expression, particularly when producing VLPs, have not despite widespread use of *Agrobacterium* to produce recombinant proteins in plants (Goulet *et al*., 2012; Grosse‐Holz *et al*., 2018; Jutras *et al*., 2020).

In this study, we combined several approaches to shed light on the complex interplay of responses taking place within plant cells transiently expressing P19 only, or co‐expressing P19 and H5^Indo^. The later (hereafter referred to as H5) corresponds to the HA protein of pandemic avian influenza virus strain H5 Indonesia and upon transient expression *in planta*, it leads to the formation of VLPs. Our results show that *Agrobacterium*‐mediated expression of these foreign proteins results in downregulation of chloroplast‐related genes (CRGs). Transient protein expression also resulted in the activation of generic defence responses, including lignification and upregulation of genes involved in oxidative stress and systemic acquired resistance (SAR). Importantly, activation levels of commonly induced pathways were higher upon H5 expression. In contrast, the activation of other molecular responses was more specific to the recombinant protein expressed, including salicylic acid (SA) responses and increased expression of cytosolic heat shock proteins (HSPs) by P19 expression. Likewise, H5 expression resulted in the modulation of lipid metabolism, of lipid distribution within membranes and in the activation of oxylipin‐related responses. Our results thus show that VLP production in *N. benthamiana* triggers a unique molecular signature that affects plant cells through enhanced expression of genes commonly involved in wounding or herbivory stresses. They also suggest that plant cells producing VLPs experience signal crosstalk, with SA‐dependent responses induced by P19 at least partially antagonized by H5 expression, perhaps due to lower P19 expression, oxylipin‐related signalling generated by H5 or a combination of both effects. Better understanding of plant responses to foreign protein expression will help to improve molecular farming techniques, as exemplified here with the exogenous application of ascorbic acid (AsA), an antioxidant that inhibited activation of plant immune responses and that improved biomass quality during VLP expression.

## Results

### Stress symptoms and HA protein expression

**Figure 1:**
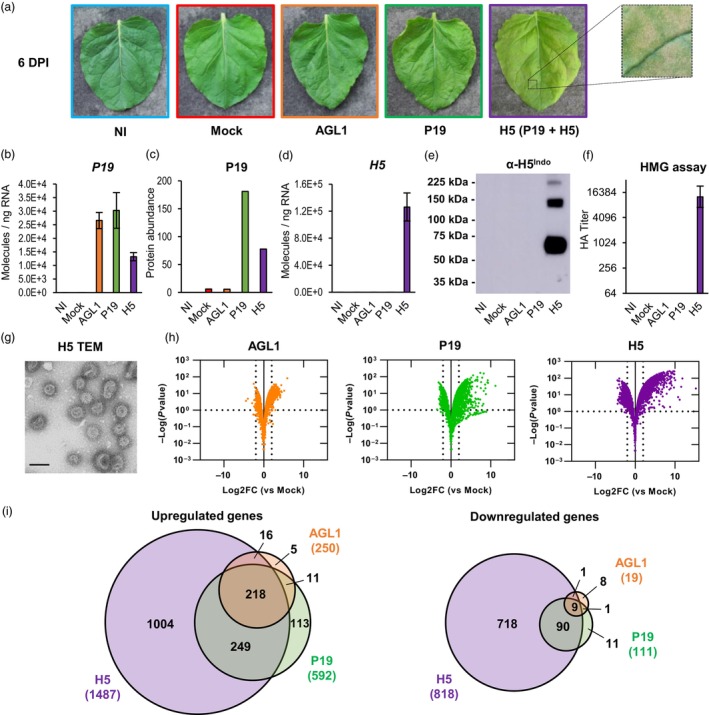
Stress symptoms and analysis of biomass used for transcriptomics. (a) Stress symptoms observed on representative leaves from each condition harvested at 6 DPI. A magnified leaf section highlights necrotic flecking seen when expressing H5. (b) Expression of recombinant gene *P19* as measured by RTqPCR at 6 DPI. Results are expressed in numbers of molecules per ng of RNA. (c) Abundance of the P19 protein as measured by iTRAQ proteomics. Results are expressed in total numbers of P19 peptides detected at 6 DPI. (d) Expression of recombinant gene *H5* as measured by RTqPCR. (e) Western blot confirming H5 protein accumulation. (f) Hemagglutination (HMG) assay confirming HA protein activity (g) Transmission electron microscopy (TEM) confirming production of influenza VLPs. Scale bar equals 100 nm. (h) Volcano plots depicting global transcriptional changes at 6 DPI after AGL1 infiltration (left panel), P19 expression (middle panel) or P19 and H5 co‐expression (right panel). Dashed lines represent expression thresholds: Log2FC ≥ 2 or ≤−2 and padj <0.1. (i) Venn diagrams depicting up‐ and down‐regulated genes after pairwise comparisons: AGL1 *versus* Mock, P19 *versus* Mock and H5 *versus* Mock. Circle size is proportional to the number of significantly regulated genes. Genes specific to AGL1 samples are shown in orange. Genes specific to P19 samples are shown in green. Genes specific to H5 samples are shown in purple. Diagram intersects show genes common to more than one condition. Condition names are as follows: NI, non‐infiltrated leaves; Mock, leaves infiltrated with buffer only; AGL1, leaves infiltrated with *Agrobacterium* strain AGL1 that carry a binary vector control; P19, leaves infiltrated with AGL1 and expressing P19 only; H5, leaves infiltrated with AGL1 and co‐expressing P19 and H5.

The effects of *Agrobacterium* infiltration, P19 expression and production of VLPs in *N. benthamiana* were characterized using multiple approaches. These first included macroscopic evaluation of stress symptoms on representative leaves of each condition harvested 6 days post‐infiltration (DPI). Using non‐infiltrated (NI) leaves as a baseline, no obvious effect was visible on leaves infiltrated only with buffer (Mock) or infiltrated with *Agrobacterium* strain AGL1 carrying a binary vector control (AGL1; Figure 1a). For AGL1‐infiltrated leaves only expressing P19, yellowish discoloration of the leaf blade was seen, suggesting light chlorosis activation. No evidence of plant cell death was denoted on P19 leaves. In comparison, leaves co‐expressing P19 and H5 showed more pronounced chlorosis, as well as greyish necrotic flecking suggesting early stage of plant cell death activation (see magnified section of the H5 leaf; Figure 1a).

To assess expression of recombinant gene *P19* at 6 DPI, real‐time quantitative polymerase chain reaction (RTqPCR) was performed. For NI and mock‐infiltrated samples, results confirmed the lack of *P19* gene expression (Figure 1b). For AGL1 and P19 samples, similar abundance of *P19* transcripts was observed. While confirming expression of the VSR gene in P19 samples, these results also indicated that *P19* transcripts were produced in AGL1 samples. The latter were obtained following infiltration of agrobacteria that carried a frameshifted version of *P19*, which does not impair transcription but provokes early termination of protein translation. In H5 samples, expression of the *P19* gene was also detected, however co‐expression of *H5* apparently reduced the number of *P19* transcripts to about half of what was observed in P19 samples (Figure 1b). Using proteomics data (see the Large‐scale proteomics section below), we also monitored accumulation levels of P19 in the various conditions tested (Figure 1c). While the P19 protein was absent in NI samples, trace amounts were detected in Mock and AGL1 samples, perhaps due to the high sensitivity of this assay. For AGL1 samples, results nonetheless confirmed efficacy of the frameshift mutation at preventing P19 translation. In line with the levels of *P19* transcripts (Figure 1b), significant accumulation of the P19 protein was detected in P19 and H5 samples, with about half of the protein abundance seen in H5 samples compared to P19 samples (Figure 1c).

To assess expression of recombinant gene *H5* at 6 DPI, RTqPCR was again employed. For NI, Mock, AGL1 and P19 samples, no expression of *H5* was detected (Figure 1d). On the other hand, recombinant gene *H5* was highly expressed in H5 samples. When comparing the numbers of *P19* and *H5* transcript molecules per ng of RNA, results showed that the latter was expressed at much higher level (compare scales from Figure 1d,b). This was consistent with predicted strength of the promoters used to drive recombinant gene expression, namely a *plastocyanin* promoter for *P19* and a *2X35S* promoter for *H5* (see the Materials and methods section). To confirm accumulation of recombinant protein H5, a western blot was performed using an antibody specific to the HA protein of influenza virus strain H5 Indonesia (α‐H5^Indo^). Results showed that the H5 protein could only be detected in H5 samples (Figure 1e). Without glycosylation, predicted molecular weight of the full‐length H5 protein is ~65 kDa, and ~62.5 kDa after removal of the signal peptide. Results from the western blot thus indicate that most of the H5 protein was in its monomeric form and no protein clipping was denoted (Figure 1e). Despite use of reducing agent and denaturing conditions for electrophoresis, larger bands corresponding to H5 protein dimers and timers were also visible. In line with results from the western blot, hemagglutination (HMG) assay indicated that HA activity was only measurable in samples expressing H5 (Figure 1f). HA activity also confirmed that the recombinant protein H5 accumulated *in planta* was still active against red blood cell receptors following its extraction from the leaf tissues (Matrosovich *et al*., 2015). Using biomass expressing H5, VLPs were then partially purified as described previously (D'Aoust et al., 2008). Transmission electron microscopy (TEM) of the purified product confirmed presence of complex structures corresponding to VLPs in both size and morphology, including a lipid membrane covered with spikes that closely resemble those of true influenza virions (Figure 1g). Overall, molecular analyses confirmed that collected plant biomass was suitable for downstream analyses, including evaluation of the effects of foreign protein expression on the plant transcriptome.

### Effects of foreign protein expression on the *N. benthamiana* leaf transcriptome

An RNAseq analysis was conducted with RNA from the tissues described above. Using the Mock treatment as a control (impact of the infiltration without *Agrobacterium*), pairwise comparisons were thus performed. To be considered significantly regulated, genes had to fulfil the following criteria: Log2 of the expression fold change value (Log2FC) ≥ 2 or ≤−2 and adjusted *P*‐value (padj) < 0.1 (corresponding to a false discovery rate below 10%). Based on these parameters, volcano plots of all sequenced genes (Figure 1h) and Venn diagrams of up‐ and down‐regulated genes (Figure 1i) were created. For each section of Venn diagrams, the unsorted list of up‐ and down‐regulated genes is available in the Table S1. At 6 DPI, results showed that AGL1 infiltration resulted in the upregulation of 250 genes and in the down‐regulation of 19 genes (Figure 1i). These numbers were lower than those observed following expression of P19 only, or co‐expression of P19 and H5 (Figure 1h,i). Venn diagrams also revealed that upregulated genes uniquely identified in AGL1 samples only accounted for 2% of all upregulated genes in this condition (5/250). On the other hand, down‐regulated genes uniquely identified in AGL1 samples accounted for 42% of the total (8/19), however this number was still low considering the small size of this gene subset (Figure 1i). In other words, gene sets from AGL1 samples largely overlapped with those of the P19 and H5 samples. In light of this, the Mock treatment was kept as control, but focus was given to P19 and H5 samples for subsequent RNAseq comparisons and confirmation of gene regulation by RTqPCR.

When comparing P19 and H5 samples, RNAseq revealed that co‐expression of the two proteins resulted in up‐ or down‐regulation of more genes than the expression of P19 only (Figure 1h,i). In addition, most genes with altered expression were specific to H5 samples, suggesting that expression of the HA protein induces a unique molecular signature in transformed plant cells. For gene sets that overlapped between P19 and H5 samples, extent of the gene up‐ or down‐regulation was also generally higher in H5‐expressing samples (Table S1). Globally, transcriptional changes were thus consistent with intensity of the stress symptoms, which were also increased for H5 samples (Figure 1a).

### Down‐regulation of chloroplast‐related genes

**Figure 2:**
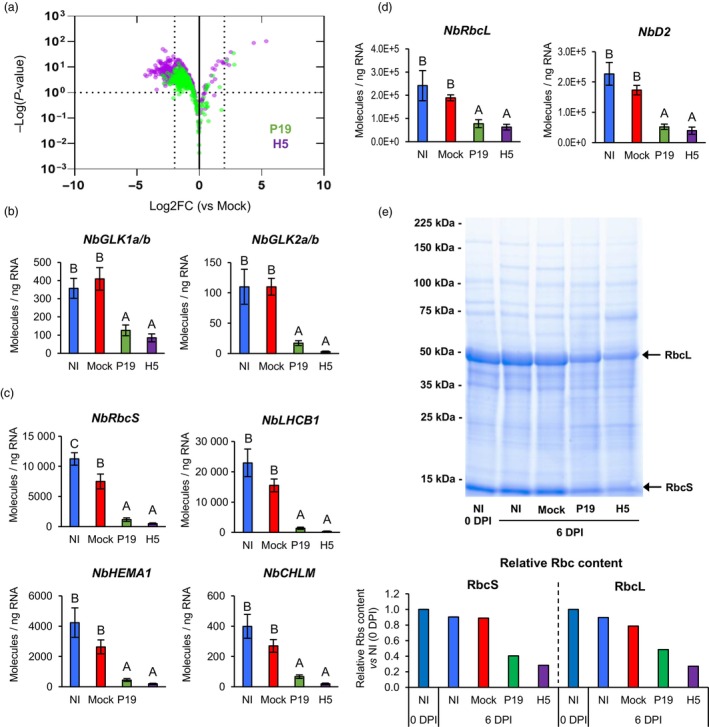
Down‐regulation of chloroplast‐related genes and decreased RuBisCO content. (a) Volcano plot depicting impact of P19 expression (green) or P19 and H5 co‐expression (purple) on CRGs at 6 DPI. Comparisons of RNAseq data were made using the Mock treatment as a control. Dashed lines represent expression thresholds: Log2FC ≥ 2 or ≤−2 and padj <0.1. Expression of *NbGLK* genes (b), of nuclear genes encoding chloroplast‐localized proteins (c) and of genes located on the chloroplast genome (d) as measured by RTqPCR at 6 DPI. Results are expressed in numbers of molecules per ng of RNA. Groups that do not share the same letter are statistically different. (e) Total protein extracts from various conditions harvested at 0 or 6 DPI. Following SDS‐PAGE analysis, gel was stained with Coomassie blue. Arrows highlight RuBisCO small and large subunits (RbcS and RbcL, respectively). Underneath bar graph depict relative RbcS and RbcL content as measured by densitometry. Rbc subunit content from leaves harvested prior to infiltration (0 DPI) were arbitrarily set at one‐fold. Condition names are as follows: NI, non‐infiltrated leaves; Mock: leaves infiltrated with buffer only; P19, leaves infiltrated with AGL1 and expressing P19 only; H5, leaves infiltrated with AGL1 and co‐expressing P19 and H5.

Gene annotation suggested that several nuclear genes encoding for chloroplast‐localized proteins were repressed in P19 and H5 samples (Table S1). These included genes encoding chlorophyll‐binding or chlorophyll synthesis proteins, protein components of the photosystems, and small subunits of the ribulose‐1,5‐bisphosphate carboxylase/oxygenase (RuBisCO; Table S2). For H5 samples, the down‐regulated gene list also comprised gene model Niben101Scf06721g00011, which is annotated as a ‘Response regulator 18’ (Table S1). Actually, this gene encodes a Golden 2‐Like (GLK) protein, a class of transcription factors (TFs) that promotes expression of CRGs (Waters *et al*., 2009). Using complete gene lists from P19 and H5 pairwise comparisons, all annotated CRGs were extracted regardless of their Log2FC or padj values (Table S2). The volcano plot created using this gene subset revealed that more CRGs were repressed in P19 and H5 samples, although not meeting the minimum Log2FC threshold of −2 (Figure 2a). The volcano plot also highlighted that for most the CRGs repressed, extent of the down‐regulation was higher in H5 samples compared to P19 samples (Figure 2a). The analysis also revealed two additional *GLK* genes that were repressed in P19 and H5 samples, although these did not meet the minimum Log2FC threshold initially established (Table S2). Also annotated as ‘Response regulators’, the down‐regulation of these genes suggests that CRG expression was broadly repressed by foreign protein expression. Indeed, the small number of down‐regulated genes in AGL1 samples (Figure 1i and Table S1) suggests that at 6 DPI, it is recombinant protein expression rather than agroinfiltration that results in down‐regulation of CRGs.

To confirm down‐regulation of CRGs, RTqPCR was performed. Tested candidates first included two pairs of closely related *GLK* genes (Figure 2b). Also analysed, were several nuclear genes encoding chloroplast‐localized proteins (Figure 2c). These were Niben101Scf02381g04022 (*NbRbcS*) that encodes a RuBisCO small subunit, Niben101Scf08088g04018 (*NbLHCB1*) that encodes a light‐harvesting chlorophyll a/b binding protein, Niben101Scf03068g00024 (*NbHEMA1*) that encodes a glutamyl‐tRNA reductase involved in chlorophyll biosynthesis and Niben101Scf03548g02031 (*NbCHLM*) that encodes a magnesium‐protoporphyrin IX methyltransferase also involved in chlorophyll synthesis. Primers specific to genes located on the chloroplast genome were also employed, and these targeted Niben101Scf00173g09004 (*NbRbcL*) that encodes a RuBisCO large subunit and Niben101Scf06964g00007 (*NbD2*) that encodes one of the photosystem II reaction centre proteins (Figure 2d). In all cases examined, RTqPCR confirmed similar or slightly lower transcript levels when comparing NI and Mock samples. In contrast, expression of CRGs was significantly reduced in P19 and H5 samples compared to NI and Mock controls. Further indication of altered chloroplast function came from the analysis of total protein extracts visualized after sodium dodecyl sulfate‐polyacrylamide gel electrophoresis (SDS‐PAGE) and Coomassie blue staining of the gel (Figure 2e). At 6 DPI, NI and Mock samples displayed similar levels of both RuBisCO subunits. These levels were also similar to those observed in NI samples harvested prior to infiltration (0 DPI). For P19 and H5 samples at 6 DPI, levels of both RuBisCO subunits had clearly decreased compared to NI and Mock controls, with a higher effect when the H5 protein was expressed.

### Upregulation of HSP and chaperone genes following P19 expression

**Figure 3:**
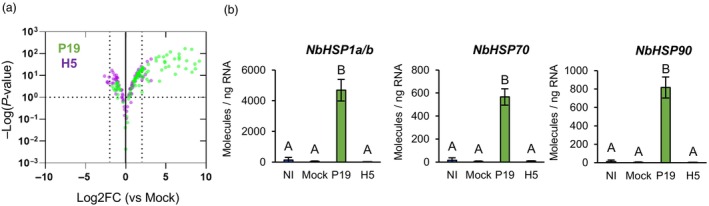
Upregulation of *HSP* and chaperone genes by P19 expression. (a) Volcano plot depicting impact of P19 expression (green) or P19 and H5 co‐expression (purple) on *HSP* and chaperone genes at 6 DPI. Comparisons of RNAseq data were made using the Mock treatment as a control. Dashed lines represent expression thresholds: Log2FC ≥ 1 or ≤−1 and padj <0.1. (b) Expression of *HSP* genes as measured by RTqPCR. Results are expressed in numbers of molecules per ng of RNA. Groups that do not share the same letter are statistically different. Condition names are as follows: NI, non‐infiltrated leaves; Mock, leaves infiltrated with buffer only; P19, leaves infiltrated with AGL1 and expressing P19 only; H5, leaves infiltrated with AGL1 and co‐expressing P19 and H5.

RNAseq revealed that 113 genes were specifically upregulated in P19 samples (Figure 1I). Among these genes, 36 (~32%) encoded cytosolic HSPs or other types of molecular chaperones (Tables S1 and S3). Using whole gene lists from the P19 and H5 pairwise comparisons, *HSP* and chaperone genes were extracted regardless of their Log2FC or padj values (Table S3). The volcano plot created using this gene subset highlighted specificity of the response in P19 samples (Figure 3a), despite co‐expression of the silencing suppressor in H5 samples (Figure 1c). As expression of only three *HSP* genes was slightly induced in AGL1 samples (Table S1), results suggest that it was P19 expression that drove this response. To confirm this particular upregulation pattern, primers targeting the highly induced *HSP* genes Niben101Scf04040g09011 (*NbHSP1a*) and Niben101Scf10306g00024 (*NbHSP1b*), as well as Niben101Scf04490g00001 (*NbHSP70*) and Niben101Scf03114g03011 (*NbHSP90*) were designed. In all cases, RTqPCR confirmed strong upregulation of *HSP* genes in P19 samples compared to the other conditions, including H5 samples (Figure 3b).

### Upregulation of lipid‐related genes

**Figure 4:**
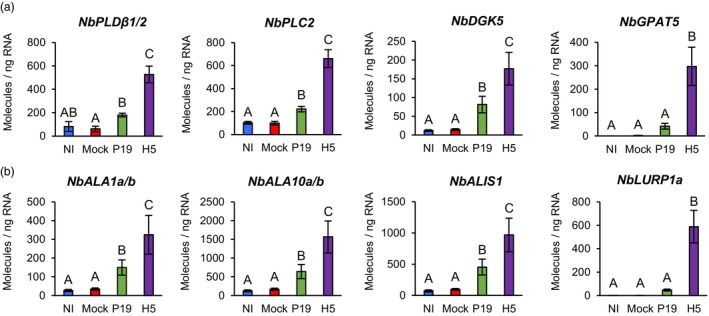
Expression of lipid‐related genes. Expression of genes involved in lipid metabolism and signalling (a), or in distribution of lipids within membranes (b) as measured by RTqPCR at 6 DPI. Results are expressed in numbers of molecules per ng of RNA. Groups that do not share the same letter are statistically different. Condition names are as follows: NI, non‐infiltrated leaves; Mock, leaves infiltrated with buffer only; P19, leaves infiltrated with AGL1 and expressing P19 only; H5, leaves infiltrated with AGL1 and co‐expressing P19 and H5.

The secretion of HA proteins requires the endomembrane system of plant cells, while lipid envelope of the VLPs is derived from the plant cell PM (D'Aoust *et al*., 2008). Consistent with this, RNAseq identified several lipid‐related genes that were induced, including those encoding two closely related phospholipases D (PLDs), one phospholipase C (PLC) and two diacylglycerol kinases (DGKs; Tables S1 and S4). In all cases, genes were induced in P19 samples, however upregulation levels were higher in H5 samples. While PLDs convert structural phospholipids into phosphatidic acid (PA), PLCs produce soluble inositol 1,4,5‐trisphosphate and diacyl glycerol (DAG) that remains in membranes, but can be converted to PA by DGKs (Canonne *et al*., 2011). The *PLD* genes identified were Niben101Scf02465g00004 (*NbPLDβ1*) and Niben101Scf16022g04010 (*NbPLDβ2*), which are most closely related to *PLDβ1* of *Arabidopsis thaliana*. The latter promotes pathogen‐induced production of jasmonic acid (JA), which in turn represses signalling mediated by SA (Zhao *et al*., 2013). The *PLC* gene identified was Niben101Scf02221g00009 (*NbPLC2*), which is most similar to Arabidopsis *PLC2* that promotes production of reactive oxygen species (ROS) during immunity (D'Ambrosio *et al*., 2017). The most highly induced *DGK* gene was Niben101Scf06654g02004 (*NbDGK5*), a close homologue of *NtDGK5* that promotes oxidative stress in *Nicotiana tabacum* (tobacco; Cacas *et al*., 2017). To confirm RNAseq results, RTqPCR was carried out with primers specific to *NbPLDβ1/2*, *NbPLC2* and *NbDGK5*. In all cases, results confirmed slight upregulation in P19 samples compared to NI and Mock controls, and a significantly higher gene induction in H5 samples (Figure 4a). RNAseq also revealed strong and in this case H5‐specific upregulation of the gene Niben101Scf10067g02017 (Tables S1 and S4), which encodes a putative glycerol‐3‐phosphate acyltransferase (GPAT). This class of enzymes converts glycerol‐3‐phosphate into lysophosphatidic acid (LPA). RTqPCR confirmed that this gene, herein termed *NbGPAT5*, was highly induced in a manner dependent on H5 expression (Figure 4a). Taken together, our results suggests that H5 protein expression favours the accumulation of PA and LPA, changes that could be due to an increased demand for certain lipid species associated to HA protein secretion, and/or budding of the VLPs.

Under standard conditions, the PM is characterized by an asymmetric distribution of its lipid components and the alteration of this asymmetry affects PM curvature (van Meer *et al*., 2008). Modulation of lipid asymmetry is thus an important feature for vesicle‐mediated secretion, in addition of being reported during host‐virus interactions and activation of plant cell death (López‐Marqués *et al*., 2014). In mammalian cells, lipid asymmetry is controlled by proteins that flip lipids from one membrane leaflet to the other, including flippases and scramblases (Devaux *et al*., 2006). In Arabidopsis, P4 ATPases (ALAs) have been shown to work as flippases (López‐Marqués *et al*., 2014), while proteins of the DUF567 family have structural features reminiscent of lipid scramblases (Bateman *et al*., 2009). RNAseq revealed that *ALA* homologues (*NbALAs*) were induced in P19 samples, but that upregulation was higher in H5 samples (Tables S1 and S4). Among induced *NbALAs* were close relatives of Arabidopsis *ALA10* that internalizes exogenous phospholipids across the PM (Poulsen *et al*., 2015). Also induced were homologues of *ALA3* and *ALA1*, which encode for proteins that localize to the Golgi apparatus and PM, respectively (López‐Marqués *et al*., 2012; Poulsen *et al*., 2008). ALA3 and ALA1 also interact with ALA‐interacting subunits (ALISs), which contribute to their sub‐cellular localization and activity. A homologue of Arabidopsis *ALIS1* was also induced in P19 and H5 samples, with higher upregulation level again observed for the latter (Tables S1 and S4). For Niben101Scf02167g00009 (*NbALA1a*) and Niben101Scf21069g00002 (*NbALA1b*), as well as Niben101Scf03029g01010 (*NbALA10a*) and Niben101Scf00465g02013 (*NbALA10b*), RTqPCR confirmed significantly higher expression in H5 samples compared to P19 samples (Figure 4b). A similar expression pattern was also observed for Niben101Scf02639g03015 (*NbALIS1*).

Interestingly, RNAseq also revealed strong, and in most cases H5‐specific, induction of genes homologous to Arabidopsis *late upregulated in response to Hyaloperonospora parasitica 1* (*LURP1*; Tables S1 and S4). While required for immunity mediated by resistance proteins (Knoth and Eulgem, 2008), *LURP1* encodes a protein of the DUF567 family and thus possess structural features reminiscent of lipid scramblases. For Niben101Scf00819g08011 (*NbLURP1a*), RTqPCR confirmed strong and H5‐specific gene upregulation (Figure 4b). Again, these transcriptional changes may be needed to alter membrane composition and architecture during HA protein secretion and/or budding of the VLPs.

### Upregulation of oxidative stress‐related genes

**Figure 5:**
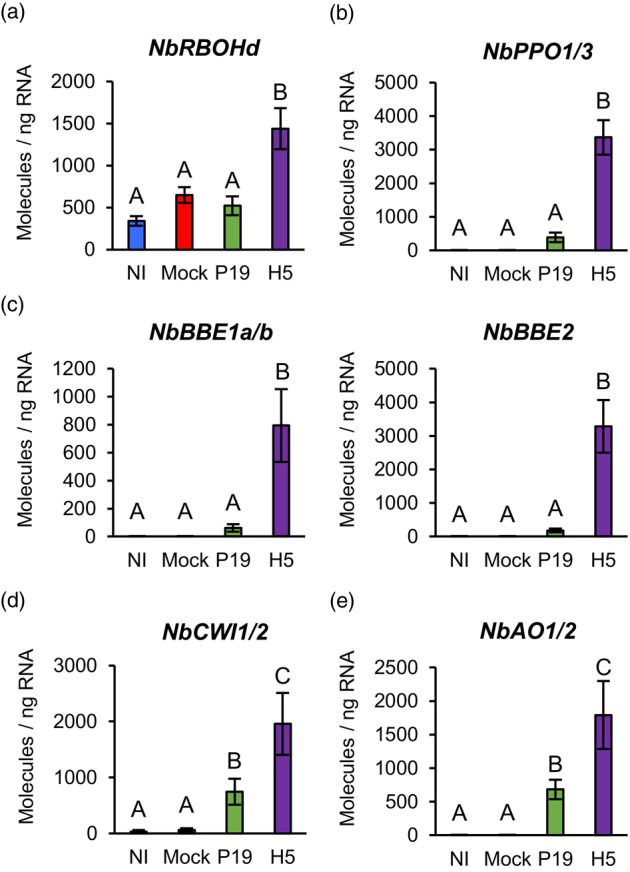
Expression of genes linked to the activation of oxidative stress. Expression of genes encoding NADPH oxidase (a), PPOs (b), secreted carbohydrate oxidases of the BBE family (c), CWIs (d) or secreted AOs (e) as measured by RTqPCR at 6 DPI. Results are expressed in numbers of molecules per ng of RNA. Groups that do not share the same letter are statistically different. Condition names are as follows: NI, non‐infiltrated leaves; Mock, leaves infiltrated with buffer only; P19, leaves infiltrated with AGL1 and expressing P19 only; H5, leaves infiltrated with AGL1 and co‐expressing P19 and H5.

In plant cells, membrane respiratory burst oxidase homologues (RBOHs) are a major source of ROS (Møller *et al*., 2007), with Arabidopsis RBOHd and RBOHf reported to be involved in plant immunity (Torres *et al*., 2002). At 6 DPI, RNAseq showed that both Niben101Scf02581g04013 (*NbRBOHd*) and Niben101Scf10840g01010 (*NbRBOHf*) were induced in a manner largely dependent on H5 protein expression (Tables S1 and S5). For *NbRBOHd*, this observation was confirmed by RTqPCR (Figure 5a). ROS are also produced by electron transport chains from stressed mitochondria and chloroplasts, as well as via the activity of other cellular oxidases (Van Aken and Van Breusegem, 2015). Among the most highly induced genes specific to H5 samples, RNAseq identified two closely related *polyphenol oxidases* (*PPOs*; Tables S1 and S5). Typically activated in response to wounding and herbivory, PPOs oxidize phenolic compounds that in turn react with oxygen and proteins to form ROS (Tran *et al*., 2012). Again, RTqPCR with primers specific to Niben101Scf00180g08002 (*NbPPO1*) and Niben101Scf04384g02014 (*NbPPO3*) confirmed H5 protein expression to result in strong and specific upregulation of these *PPO* genes (Figure 5b).

In response to H5 expression, several genes encoding for berberine‐bridge enzymes (BBEs) were also among the most highly induced (Tables S1 and S5). BBEs belong to a functionally diverse protein family, yet these oxidases all produce hydrogen peroxide (Daniel *et al*., 2017). BBEs have been involved in a number of cellular processes, however *NbBBE* genes that were here highly induced all encoded secreted proteins sharing homology to carbohydrate oxidases (Carter and Thornburg, 2004; Custers *et al*., 2004). This suggests that H5 expression promotes sugar oxidation in the apoplast, which may in turn contribute to the activation of oxidative stress. In line with this, RNAseq revealed that expression of genes related to sugar metabolism in the apoplast was induced in P19 samples, but even more so in H5 samples (Tables S1 and S6). These included homologues of sugar transport proteins (STPs) and cell wall invertases (CWIs) that participate in plant defence (Lemonnier *et al*., 2014; Veillet *et al*., 2016). For *BBE* genes Niben101Scf01395g03002 (*NbBBE1a*) and Niben101Scf01061g07011 (*NbBBE1b*), as well as Niben101Scf00944g01001 (*NbBBE2*), strong and H5‐specific induction was confirmed by RTqPCR (Figure 5c). RTqPCR also confirmed induction of the *CWI* genes Niben101Scf12270g02002 (*NbCWI1*) and Niben101Scf04632g03006 (*NbCWI2*) in both P19 and H5 samples, with again upregulation levels significantly higher in the latter (Figure 5d). Taken together these results suggest that foreign protein expression promotes conversion of sucrose to simple sugars in the apoplast, possibly providing energy to support local activation of plant defence. However, in H5‐expressing samples, increased accumulation of simple sugars in the apoplast may contribute to ROS production via proteins encoded by *NbBBE* genes that are strongly induced in this condition (Figure 5c).

To prevent extensive damage caused by ROS, plant cells produce antioxidant metabolites, including ascorbic acid (AsA) that is the major antioxidant of the apoplast (Pignocchi *et al*., 2006). RNAseq showed that closely related *ascorbate oxidase* (*AO*) genes were upregulated in AGL1, P19 and H5 samples, with a higher upregulation level in the latter (Tables S1 and S5). As the redox status of apoplastic AsA is negatively controlled by secreted AOs (Pignocchi and Foyer, 2003), our results suggest that upregulation of these genes also contributes to oxidative stress activation in the apoplast, especially when the H5 protein is expressed. RTqPCR with primers specific to Niben101Scf03026g01009 (*NbAO1*) and Niben101Scf22432g00001 (*NbAO2*) confirmed upregulation of these genes in P19 and H5 samples compared to controls, with again significantly higher expression level in the latter (Figure 5e).

### Lignin‐related gene expression and lignin quantification

**Figure 6:**
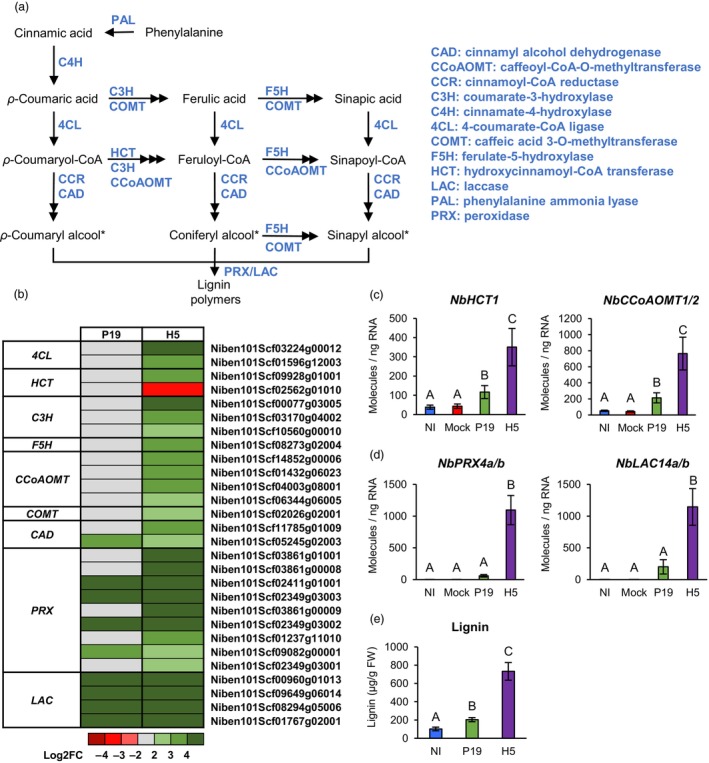
Expression of lignin‐related genes and lignin quantification. (a) Overview of the lignin biosynthesis pathway. Metabolites are shown in black, while enzymes are shown in blue. Monolignol precursors of lignin are marked with an asterisk. (b) Heat‐map depicting expression of genes involved in monolignol precursor synthesis or lignin polymerization in the cell wall at 6 DPI. Each line represents a gene shown in the Table S7. Grey indicates genes that are not differentially expressed. Green are red coloured gradients, respectively reflect extent of gene up‐ and down‐regulation, as indicated. At 6 DPI, RTqPCR confirms upregulation of monolignol synthesis genes (c), as well as genes involved in lignin polymerization (d). Results are expressed in numbers of molecules per ng of RNA. (e) Lignin accumulation at 6 DPI. Results are expressed in ug of lignin per g of biomass fresh weight (FW). Groups that do not share the same letter are statistically different. Condition names are as follows: NI, non‐infiltrated leaves; Mock, leaves infiltrated with buffer only; P19, leaves infiltrated with AGL1 and expressing P19 only; H5, leaves infiltrated with AGL1 and co‐expressing P19 and H5.

In response to pathogens, plant cells reinforce their cell wall through the coordinated deposition of polymers, including lignin (Wang *et al*., 2013). The lignin biosynthesis pathway comprises numerous classes of enzymes that catalyse synthesis and cell wall polymerization of monolignol precursors (Figure 6a). When expressing secreted VLPs, lignification poses extra challenges to extract and purify desired product from the reinforced plant cell wall. In H5 samples, RNAseq suggested specific induction or at least stronger expression, of several genes with predicted function in lignification (Tables S1 and S7). Notably, secreted *peroxidase* (*PRX*) and *laccase* (*LAC*) genes were among the most highly induced in response to H5 expression. Also upregulated were monolignol synthesis genes, including *4‐coumarate‐CoA ligases* (*4CLs*), a *hydroxycinnamoyl‐CoA transferase* (*HCT*) and *caffeoyl‐CoA O‐methyltransferases* (*CCoAOMTs*; Table S7). When displayed as a heat‐map, our results in fact showed that for H5 samples, most steps of the lignin biosynthesis pathway were represented by at least one upregulated gene (Figure 6b). To confirm these results, RTqPCR was performed on selected genes either involved in monolignol synthesis (Figure 6c), or lignin polymerization (Figure 6d). Compared to controls, results showed that genes were slightly induced by expression of P19 only, but that co‐expression of P19 and H5 resulted in significantly higher gene induction. To confirm lignin accumulation during foreign protein expression, lignin content was quantified in NI, P19 and H5 samples harvested at 6 DPI (Figure 6e). These results showed that P19 and H5 samples accumulated more lignin than the NI control, again with a much stronger effect in H5 samples.

### Salicylic acid synthesis and signalling

**Figure 7:**
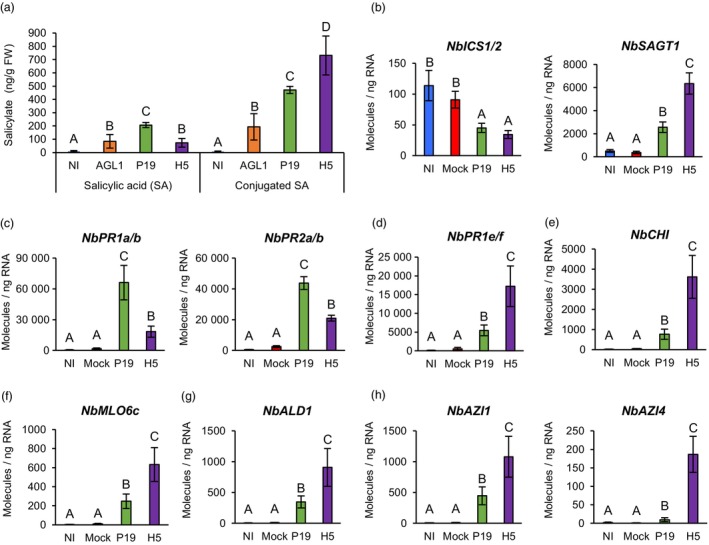
Salicylate accumulation and expression of SA‐ and SAR‐related genes. (a) Accumulation of SA and conjugated SA at 6 DPI. Results are expressed in ng of salicylate per g of biomass fresh weight (FW). RTqPCR confirms that salicylate accumulation is coupled to the expression of SA regulatory genes (b) and of SA response genes (c). RTqPCR was also performed to assess expression of different SAR response genes (d, e and f) and of SAR regulatory genes (g and h). RTqPCR results are expressed in numbers of molecules per ng of RNA. Groups that do not share the same letter are statistically different. Condition names are as follows: NI, non‐infiltrated leaves; Mock, leaves infiltrated with buffer only; AGL1, leaves infiltrated with *Agrobacterium* strain AGL1 that carry a binary vector control; P19, leaves infiltrated with AGL1 and expressing P19 only; H5, leaves infiltrated with AGL1 and co‐expressing P19 and H5.

To shed light on stress hormone signalling during foreign protein expression, SA was quantified in NI, AGL1, P19 and H5 samples. At 6 DPI, NI samples showed barely detectable levels of SA, while significantly higher SA accumulation was observed in P19 samples compared to AGL1 and H5 samples (Figure 7a). From these results, we deduced that AGL1 infiltration induces moderate accumulation of SA and that expression of P19 increases this response. In H5 samples, which co‐express P19 and H5 proteins, P19‐mediated accumulation of SA appeared to be compromised. This was perhaps caused by lower P19 levels in H5 samples (Figure 1c), because HA‐induced signalling interfered with this process, or a combination of both effects. In AGL1, P19 and H5 samples, it was also found that most salicylates were in a conjugated form, with highest levels detected in H5 samples compared to P19 and even more so AGL1 samples (Figure 7a).

In plants, SA is produced via two independent pathways (Chen *et al*., 2009). In *N. benthamiana*, stress‐induced production of SA however depends on the expression of *isochorismate synthase* (*ICS*) genes (Catinot *et al*., 2008). RNAseq indicated that Niben101Scf00593g04010 (*NbICS1*) was repressed in both P19 and H5 samples (Tables S1 and S8). RTqPCR targeting *NbICS1* and its close homologue Niben101Scf05166g06006 (*NbICS2*) confirmed reduced *ICS* gene expression in both P19 and H5 samples compared to controls (Figure 7b). This suggests that P19‐induced accumulation of SA (Figure 7a) is either not dependent on *NbICS* gene upregulation, or that this upregulation occurred before harvesting at 6 DPI. Alternatively, this response may be mediated through the other SA biosynthesis route, although *phenylalanine ammonia lyase* (*PAL*) genes, which encode the first enzyme of this pathway, were not induced in the tested conditions.

In Arabidopsis, decreases in SA levels have been associated with transcriptional repression of *ICS1* and increased SA storage caused by upregulation of the gene *SA glucosyl transferase 1* (*SAGT1*; Zheng *et al*., 2012). Protein product of the latter converts SA into inactive conjugated forms that are stored for later use (Dean and Delaney, 2008). In *N. benthamiana*, Niben101Scf05415g00003 is the closest homologue of *SAGT1*. Here termed *NbSAGT1*, RNAseq showed that this gene was induced in P19 samples, but that upregulation level was higher in H5 samples (Tables S1 and S8). This expression pattern was confirmed by RTqPCR (Figure 7b), indicating that transcriptomics data correlated with detected levels of conjugated SA (Figure 7a).

To further characterize SA signalling, expression of *Pathogenesis Related 1* (*PR1*) genes was investigated. The genome of *N. benthamiana* comprises at least 20 *PR1‐like* genes, with Niben101Scf01999g07002, Niben101Scf00107g03008, Niben101Scf03376g03004 and Niben101Scf13926g01014 being the most similar to *AtPR1* (At2g14610; Figure S1). This Arabidopsis homologue is the most widely employed as a marker of SA signalling. The first three of these *N. benthamiana* genes, respectively termed *NbPR1a*, *NbPR1b* and *NbPR1c*, were identified by RNAseq, with expression levels higher in P19 samples compared to AGL1 and H5 samples (Tables S1 and S8). This pattern thus correlated with respective SA accumulation of AGL1, P19 and H5 samples (Figure 7a). RTqPCR with primers specific to *NbPR1a* and *NbPR1b* confirmed higher upregulation of these genes in P19 samples compared to H5 and furthermore control samples (Figure 7c). Interestingly, similar expression patterns were observed for *β‐1,3‐glucanase* genes of the *Pathogenesis Related 2* (*PR2*) family, which are also often employed as markers of SA signalling (Durrant and Dong, 2004). In these cases, RNAseq revealed similar induction levels in AGL1 and P19 samples, while expression was lower in H5 samples (Tables S1 and S8). For Niben101Scf01001g00003 (*NbPR2a*) and Niben101Scf01001g00005 (*NbPR2b*), RTqPCR confirmed higher gene upregulation in P19 samples compared to H5 samples and furthermore control conditions (Figure 7c).

### Expression of SAR‐related genes

Close examination of RNAseq data highlighted the upregulation of a second *PR1* gene subset, which interestingly behaved differently compared to their previously described homologues (see above). In this case, Niben101Scf04053g02007 (*NbPR1e*), Niben101Scf04053g02006 (*NbPR1f*) and Niben101Scf00953g03009 (*NbPR1g*) were induced in AGL1 and P19 samples, but expression was more pronounced in H5 samples (Tables S1 and S8). Importantly, these three genes were found to be closely related as they encode structurally distinct PR1 proteins that possess a short C‐terminal extension (CTE) following the conserved CAPE1 peptide of PR1 proteins (Figure S1; Chen *et al*., 2014). RTqPCR with primers specific to *NbPR1e* and *NbPR1f* confirmed the distinct transcriptional behaviour of these *PR1* genes, with highest expression levels detected in H5 samples compared to P19 or control samples (Figure 7d).

Enhanced *PR1* gene expression is not only associated to SA signalling, but also to SAR (Durrant and Dong, 2004). Expression of SAR‐related genes was thus examined, including *N. benthamiana* homologues of the *Pathogenesis Related 3* (*PR3*) gene *Chitinase* (*CHI*) and *Mildew Resistance Locus O 6 (MLO6*) of Arabidopsis (Riedlmeier *et al*., 2017). RNAseq revealed that several *PR3* genes were induced in AGL1, P19 and H5 samples, with higher upregulation levels seen in the latter (Tables S1 and S8). This included Niben101Scf02171g00007 (*NbCHI*), the closest homologue of *CHI* from Arabidopsis. For *MLO6* homologues, several candidates were specifically induced in H5 samples, while others were induced in both P19 and H5 samples, again with higher transcript levels in the latter (Tables S1 and S8). This was, for instance, the case for Niben101Scf07792g02034 (*NbMLO6c*), the closest homologue of *MLO6* from Arabidopsis. As for *NbPR1e* and *NbPR1f* (Figure 7d), RTqPCR confirmed upregulation of *NbCHI* (Figure 7e) and *NbMLO6c* (Figure 7f) in P19 samples compared to controls, however induction was significantly higher in H5 samples.

To further confirm that SAR is part of the response to H5 protein expression, RNAseq data was searched for SAR regulatory genes, including homologues of Arabidopsis *AGD2‐Like Defense 1* (*ALD1*; Song *et al*., 2004) and *Azelaic Acid‐Induced 1* (*AZI1*; Cecchini *et al*., 2015). While Niben101Scf04547g02001 (*NbALD1*) was induced in AGL1 and P19 samples, upregulation level was higher in H5 samples (Tables S1 and S8). For *AZI1* homologues, Niben101Scf13429g02004 (*NbAZI1*) and Niben101Scf13429g03011 (*NbAZI5*) were similarly induced in AGL1 and P19 samples, while induction was higher in H5 samples. For Niben101Scf04779g01029 (*NbAZI2*), Niben101Scf09387g02004 (*NbAZI3*) and Niben101Scf07599g00019 (*NbAZI4*), enhanced expression was only detected in H5 samples (Tables S1 and S8). For *NbALD1* (Figure 7g) and *NbAZI* genes (Figure 7h), expression patterns observed by RNAseq were confirmed via RTqPCR, including specific induction of *NbAZI4* in H5 samples. Taken together, these results suggest that at 6 DPI, SAR‐related responses were induced in AGL1 and P19 samples, however activation of this pathway was stronger and broader in H5‐expressing samples. Distinct expression patterns of the two *PR1* gene subsets also suggest that PR1 proteins with a CTE are more indicative of SAR, while PR1 proteins without a CTE were as expected indicative of SA signalling.

### Oxylipin synthesis and signalling

**Figure 8:**
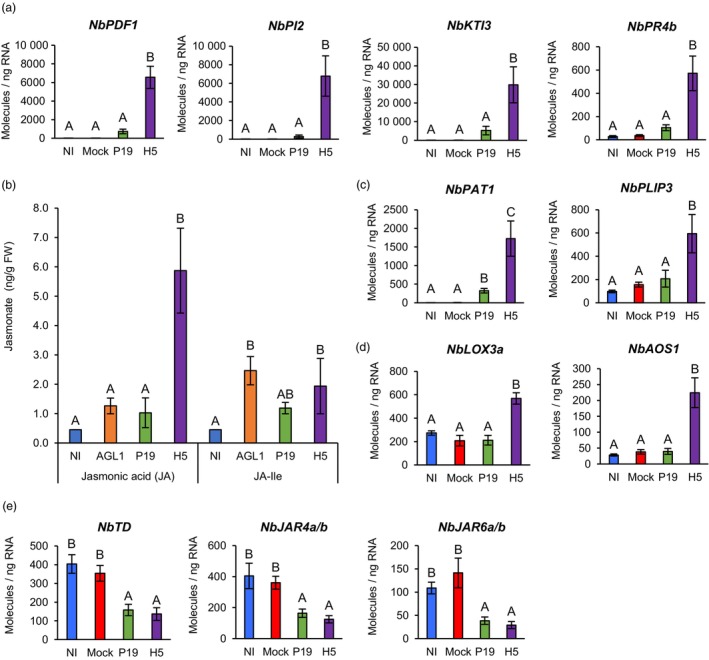
Jasmonate accumulation and expression of oxylipin‐related genes. (a) Expression of oxylipin response genes as measured by RTqPCR at 6 DPI. (b) Accumulation of JA and JA‐Ile at 6 DPI. Results are expressed in ng of jasmonate per g of biomass fresh weight (FW). RTqPCR was also performed on genes encoding PLAs (c), as well as on genes involved in JA (d) and JA‐Ile (e) biosynthesis. RTqPCR results are expressed in numbers of molecules per ng of RNA. Groups that do not share the same letter are statistically different. Condition names are as follows: NI, non‐infiltrated leaves; Mock, leaves infiltrated with buffer only; AGL1, leaves infiltrated with *Agrobacterium* strain AGL1 that carry a binary vector control; P19, leaves infiltrated with AGL1 and expressing P19 only; H5, leaves infiltrated with AGL1 and co‐expressing P19 and H5.

As discussed for *PPO* genes (Figure 5b), transcriptomics suggested that wounding and herbivory response genes were induced by H5 expression. Indeed, a search of RNAseq data revealed H5‐specific induction of several genes that typically respond to these stresses, including those that encode plant defensins (PDFs), serine protease inhibitors (PIs), Kunitz trypsin inhibitors (KTIs) and cysteine PIs of the Pathogenesis Related 4 (PR4) family (annotated as ‘wound‐induced proteins’; Tables S1 and S9). For plant defensin gene Niben101Scf17290g01005 (*NbPDF1*) as well as *PI* genes Niben101Scf00294g01014 (*NbPI2*), Niben101Scf06424g00003 (*NbKTI3*) and Niben101Scf00773g08003 (*NbPR4b*), RTqPCR confirmed strong and H5‐specific upregulation (Figure 8a).

In plants, wounding and herbivory responses often depend on stress hormone JA (Wasternack and Feussner, 2018). Upregulation of genes such as *PPOs*, *PDFs* and *PIs* thus prompted evaluation of JA levels in NI, AGL1, P19 and H5 samples. While low JA levels were detected for the first three conditions, significantly higher JA accumulation was detected in H5 samples (Figure 8b). Accordingly, RNAseq revealed upregulation of many *phospholipase A* (*PLA*) genes, in a manner largely dependent on H5 protein expression (Tables S1 and S9). These included closely related Niben101Scf03309g01011 (*NbPLA1*) and Niben101Scf01228g02012 (*NbPLA2*), which encode secreted proteins. Also induced were several *patatin* (*PAT*) genes encoding proteins closely related to *N. tabacum* homologues that promote accumulation of several oxylipins, including JA (Cacas *et al*., 2005; Dhondt *et al*., 2002). Closely related Niben101Scf01076g05026 and Niben101Scf01623g13002, which encode homologues of Arabidopsis PLASTID LIPASEs (PLIPs), were also identified. Localized in chloroplasts, PLIPs also promote the accumulation of bioactive oxylipins in response to stress (Wang *et al*., 2018). RTqPCR with primers specific to the Niben101Scf06277g00007 (*NbPAT1*) and Niben101Scf01623g13002 (*NbPLIP3*) genes confirmed their upregulation, with patterns mostly specific to H5 expression (Figure 8c).

To synthesize JA, free fatty acids produced by PLAs are oxygenated by 13‐lipoxygenases (13‐LOXs), before cyclization by allene oxide synthases (AOSs) and allene oxide cyclases (AOCs). The resulting 12‐oxophytodienoic acid (OPDA) intermediate that is produced then translocates to peroxisomes where reductases and β‐oxidation cycles complete the synthesis of JA (Wasternack and Feussner, 2018). Consistent with JA accumulation in H5 samples (Figure 8b), *13‐LOX* gene Niben101Scf02749g01001 (*NbLOX3a*), *AOS* gene Niben101Scf05799g02010 (*NbAOS1*) and *OPDA reductase* (*OPR*) gene Niben101Scf00779g06009 (*NbOPR1*) were all specifically upregulated in H5 samples (Tables S1 and S9). For *NbLOX3a* and *NbAOS1*, RTqPCR confirmed H5‐specific upregulation of gene expression (Figure 8d).

To become fully active, JA needs to conjugated to isoleucine (Ile; Staswick and Tiryaki, 2004). Quantification of jasmonate‐isoleucine (JA‐Ile) revealed overall low levels of the compound, with non‐significant differences observed between NI and P19 samples, and a small but significant increase in AGL1 and H5 samples relative to NI samples but not P19 samples (Figure 8b). In tobacco, herbivore‐induced formation of JA‐Ile requires the *Jasmonate‐Resistant 4* and *6* (*JAR4/6*) genes (Wang *et al*., 2008), as well as the gene *threonine deaminase* (*TD*) that is involved in biosynthesis of Ile (Kang *et al*., 2006). At the threshold examined, *N. benthamiana* homologues from these genes were not identified as being differentially expressed by RNAseq. However, RTqPCR analyses revealed that Niben101Scf02502g14001 (*NbTD*) was significantly repressed in P19 and H5 samples compared to NI and Mock controls (Figure 8e). Similarly, primers targeting closely related Niben101Scf05584g01007 (*NbJAR4a*) and Niben101Scf22940g00002 (*NbJAR4b*), or Niben101Scf00470g00001 (*NbJAR6a*) and Niben101Scf01083g00008 (*NbJAR6b*), showed significantly reduced expression in P19 and H5 samples compared to NI and Mock controls (Figure 8e). These results were thus consistent with overall low accumulation of JA‐Ile (Figure 8b).

**Figure 9:**
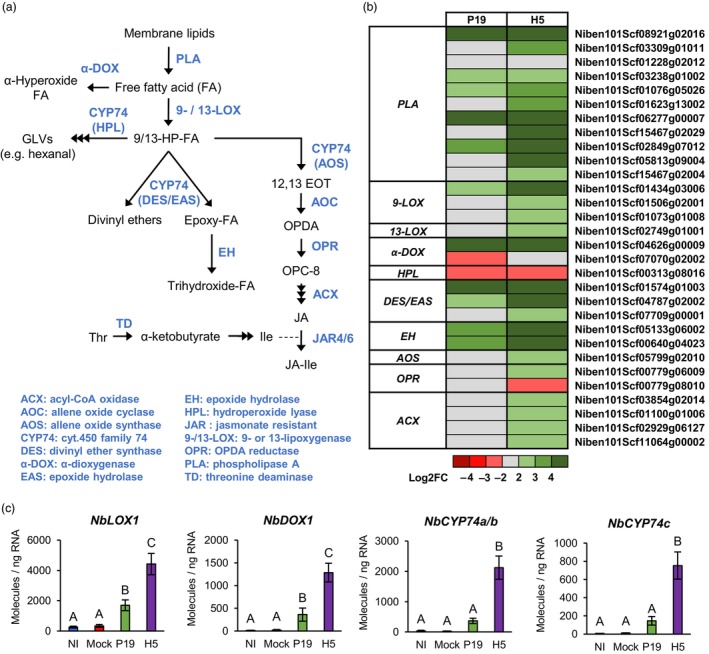
Expression of additional oxylipin regulatory genes. (a) Overview of the octadecanoid pathway. Metabolic intermediates are shown in black while enzymes are shown in blue. (b) Heat‐map depicting expression of genes involved in oxylipin metabolism at 6 DPI. Each line represents a gene shown in the Table S9. Grey indicates genes that are not differentially expressed. Green are red coloured gradients respectively reflect extent of gene up‐ and down‐regulation, as indicated. (c) Expression of genes involved in the synthesis of oxylipins other than JA as measured by RTqPCR at 6 DPI. Results are expressed in numbers of molecules per ng of RNA. Groups that do not share the same letter are statistically different. Condition names are as follows: NI, non‐infiltrated leaves; Mock, leaves infiltrated with buffer only; P19, leaves infiltrated with AGL1 and expressing P19 only; H5, leaves infiltrated with AGL1 and co‐expressing P19 and H5.

Strong H5‐specific upregulation of wounding and herbivory response genes, coupled to low JA‐Ile accumulation, suggested production of other bioactive oxylipins in response to H5 protein expression. Aside from JA and JA‐Ile, the octadecanoid pathway allows for the synthesis of other oxylipins with roles in defence (Figure 9a; Wasternack and Feussner, 2018). To shed light on which of these metabolites may be active in H5 samples, RNAseq data was searched for more oxylipin regulatory genes (Figure 9b). This revealed several candidates involved in alternative branches of the octadecanoid pathway. All identified genes were induced in a manner either specific or at least more pronounced in H5 samples (Tables S1 and S9). These included *9‐lipoxygenase* (*9‐LOX*) genes, among which Niben101Scf01434g03006 (*NbLOX1*) was the most highly induced (Figure 9b). Also identified was Niben101Scf04626g00009, an *α‐dioxygenase* (*DOX*) gene termed *NbDOX1*. In Arabidopsis, closest homologues of *NbLOX1* and *NbDOX1* work in conjugation to promote synthesis of oxylipins that activate local and systemic defences (Vicente *et al*., 2012). A number of genes encoding cytochromes P450 of family 74 (CYP74s) were also identified (Figure 9b; Tables S1 and S9). Improperly annotated as *AOS* genes, these candidates actually encode divinyl ether synthases (DESs) or epoxyalcohol synthases (EASs) that are related to, but functionally distinct from AOSs that promote JA synthesis (Figure 9a). In tobacco, NtDES1 for instance works in coordination with 9‐LOX enzymes to produce divenyl ether fatty acids involved in defence (Fammartino *et al*., 2007). Our analyses also identified Niben101Scf05133g06002 (*NbEH1*) and Niben101Scf00640g04023 (*NbEH2*), closely related *epoxide hydrolase* (*EH*) genes upregulated in AGL1 and P19 samples, but even more so in H5 samples (Figure 9b; Tables S1 and S9). Often associated to cell detoxification, EHs also act downstream of EASs to produce oxylipin diols with signalling and anti‐microbial functions (Figure 9a; Morisseau, 2013). RTqPCR analyses targeting *NbLOX1*, *NbDOX1* and *NbCYP74* genes confirmed increased expression in P19 and H5 samples compared to controls. In all cases, upregulation levels were however substantially higher in H5 samples (Figure 9c). Taken as a whole, our data suggests that VLP expression results in stronger activation of the octadecanoid pathway compared to agroinfiltration, or expression of P19 only. In addition to JA, oxylipins produced by alternative branches of the octadecanoid pathway thus appear to be actively signalling, especially in H5 samples.

### Ethylene and senescence‐related pathways

**Figure 10:**
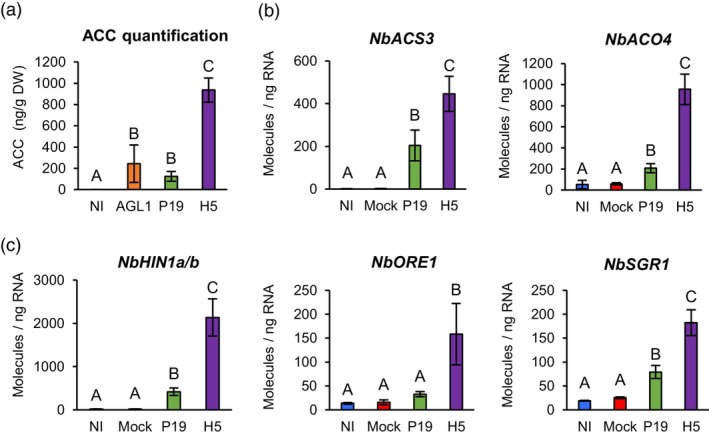
ACC accumulation and expression of ET/senescence‐related genes. (a) Accumulation of ET precursor ACC at 6 DPI. Results are expressed in ng of ACC per g of biomass dry weight (DW). RTqPCR confirms that ACC accumulation is coupled to the expression of genes involved in ET synthesis (b). RTqPCR was also performed on senescence‐related genes (c). RTqPCR results are expressed in numbers of molecules per ng of RNA. Groups that do not share the same letter are statistically different. Condition names are as follows: NI: non‐infiltrated leaves; Mock, leaves infiltrated with buffer only; AGL1, leaves infiltrated with *Agrobacterium* strain AGL1 that carry a binary vector control; P19, leaves infiltrated with AGL1 and expressing P19 only; H5, leaves infiltrated with AGL1 and co‐expressing P19 and H5.

In coordination with JA, ethylene (ET) is involved in resistance to necrotrophic pathogens, acting antagonistically with the SA pathway (Binder, 2020). To assess ET signalling in response to foreign protein expression, levels of ET precursor 1‐aminocyclopropane‐1‐carboxylate (ACC) were evaluated in NI, AGL1, P19 and H5 samples. At 6 DPI, ACC was not detected in NI samples, while AGL1 and P19 samples displayed low and not significantly different levels of ACC (Figure 10a). In contrast, H5 samples displayed significantly higher level of ACC, suggesting that ET signalling is part of the molecular response to H5 protein expression. Accordingly, RNAseq revealed upregulation of several ET biosynthesis genes, including *ACC synthases* (*ACSs*) and *ACC oxidases* (*ACOs*). While some genes were similarly induced in AGL1, P19 and H5 samples, upregulation of other candidates was more pronounced or even specific to H5 expression (Tables S1 and S10). For *ACS* gene Niben101Scf09512g03008 (*NbACS3*) and *ACO* gene Niben101Scf08039g01005 (*NbACO4*), RTqPCR confirmed significantly higher upregulation in H5 samples compared to P19 and control conditions (Figure 10b).

In addition to its roles in plant defence, ET is often associated with senescence, a form of programmed cell death linked to plant development (Binder, 2020). At 6 DPI, RNAseq indicated that several genes commonly associated with leaf senescence were induced AGL1 and P19 samples, but that upregulation levels were much higher in H5 samples (Tables S1 and S10). These included homologues of *N. tabacum asparagine synthetase* (*ASN*) genes, which are involved in remobilization of nitrogen resources during senescence (Bovet *et al*., 2019). Also identified were homologues of *N. tabacum* gene *harpin‐induced 1* (*NtHIN1*), which is induced during hypersensitive response and senescence (Takahashi *et al*., 2004). Close homologues of Arabidopsis genes *ORESARA 1* (*ORE1*) and *STAYGREEN 1* (*SGR1*) were also among identified candidates and these genes were specifically induced in H5 samples (Tables S1 and S10). While ORE1 stimulates senescence by antagonizing GLK TFs (Rauf *et al*., 2013), SGR1 promotes senescence‐related degradation of the chlorophyll pigments (Sakuraba *et al*., 2015). For Niben101Scf08020g06001 (*NbHIN1a*) and Niben101Scf04717g02003 (*NbHIN1b*), as well as Niben101Scf01277g00002 (*NbORE1*) and Niben101Scf00490g01040 (*NbSGR1*), RTqPCR confirmed higher or even specific gene upregulation in H5 samples (Figure 10c).

### Large‐scale proteomics

To investigate changes in protein abundance, a new set of NI, Mock, AGL1, P19 and H5 samples were harvested at 6 DPI. After evaluation of leaf symptoms (Figure S2A), H5 protein accumulation and HA activity were confirmed by western blotting and HMG assays, respectively (Figure S2B,C). Using total protein extracts, a proteomics analysis was then performed using the isobaric tags for relative and absolute quantitation (iTRAQ) method. Using the Mock treatment as control, pairwise comparisons were performed for AGL1, P19 and H5 samples. To be considered significantly changed in abundance, proteins had to display a Log2FC value ≥1 or ≤−1. From generated protein lists, Venn diagrams of up‐ and down‐regulated proteins were created (Figure S2D). For each diagram section, the unsorted list of up‐ or down‐regulated proteins, along with their deduced Z‐score, is available in the Table S11.

At the threshold examined, a total of 194 proteins were upregulated, including 89 (~46%) common to AGL1, P19 and H5 samples (Figure S2D). On the opposite, 75 proteins were down‐regulated, including 22 (~30%) common to all conditions. In line with the transcriptome, AGL1 infiltration altered the abundance of fewer proteins compared to P19 expression. In turn, expression of P19 altered the abundance of fewer proteins compared to P19 and H5 co‐expression. Many proteins with altered abundance also turned out to be specific to H5 samples, again highlighting induction of a unique molecular signature upon VLP expression. As for the transcriptome, abundance of proteins shared between H5 samples and other conditions (AGL1, P19 or both) was generally more affected when H5 was expressed (Table S11).

Out of the 16 proteins specifically upregulated in P19 samples (Figure S2D), half were cytosolic HSPs (Table S11). Corresponding genes had all been identified by RNAseq (Table S3). Several PIs were also upregulated, including NbPI2 in AGL1 and H5 samples, as well as NbKTI1, NbKTI3 and NbPR4b in P19 and H5 samples (Table S11). In all cases, protein accumulation was at least two times higher in H5 samples compared to AGL1 or P19 samples. Genes encoding identified PIs had also been identified by RNAseq (Table S9). Enhanced accumulation of oxylipin synthesis enzymes was also observed, including NbOPR1 and NbOPR3, that were specific to H5 samples (Table S11). While Niben101Scf00779g06009 (*NbOPR1*) had been identified in the transcriptome (Figure 9b), Niben101Scf05804g03006 (*NbOPR3*) was not, at least at the threshold examined. Oxylipin‐related proteins NbLOX1 and NbEH1 were also among upregulated proteins identified, and both accumulated to higher levels in H5 samples compared to AGL1 or P19 samples (Table S11). Again, the corresponding genes had been identified in the transcriptome (Table S9).

Among upregulated proteins, secreted enzymes linked to the activation of oxidative stress in the apoplast were also identified. These were the putative carbohydrate oxidase NbBBE2 and the lignin‐forming peroxidase NbPRX4a, which were both specific to H5 samples (Table S11). Also identified were apoplastic sugar invertases NbCWI1 and NbCWI2, as well as ascorbate oxidase NbAO2. These proteins were identified in AGL1, P19 and H5 samples, however with greater accumulation levels in the latter (Table S11). For all secreted enzymes, protein accumulation pattern matched with the expression profile of corresponding genes in the transcriptome.

Consistent with activation of plant immunity, upregulation of PR proteins was also detected, including SA markers NbPR2a and NbPR2b. In agreement with SA accumulation levels (Figure 7a) and expression of corresponding *PR2* genes (Figure 7c), these proteins accumulated to higher levels in AGL1 and P19 samples compared to H5 samples (Table S11). Again, this supports the idea that SA‐mediated signalling was induced by agroinfiltration and furthermore by P19 expression, but that this response was partially antagonized in H5 samples. Peptides belonging to NbPR1e and NbPR1f, which harbour a CTE (Figure S1), were also identified by proteomics. In both cases, protein accumulation was higher in H5 samples compared to AGL1 or P19 samples (Table S11). This pattern was also observed for NbCHI, a marker of SAR. As expression profiles from *NbPR1e*, *NbPR1f* and *NbCHI* (Figure 7d,e) matched protein accumulation patterns, our results support the idea that PR1 homologues with a CTE better reflect activation of SAR, and that this pathway is part of the plant response to VLP expression.

### Proteins linked to the unfolded protein response

As described above, proteomics data generally correlated with results from the transcriptome. Interestingly, proteomics also highlighted accumulation of proteins involved in the unfolded protein response (UPR). This pathway allows cells to cope with increasing needs in protein secretion, in addition of preventing deleterious effects provoked by accumulation of misfolded proteins in the endoplasmic reticulum (ER; Duwi Fanata *et al*., 2013). Agroinfiltration coupled to enforced expression of a complex secreted protein such as H5 would, for instance, be expected to cause ER stress and thus to activate the UPR. Accordingly, proteomics identified protein disulfide isomerases (PDIs) with ER retention signals, as well as ER‐resident chaperones of the calreticulin (CRT) and binding immunoglobulin protein (BiP) families (proteins marked with an asterisk in the Table S11). Intriguingly, the corresponding UPR genes were not identified in the transcriptome at 6 DPI. This suggests that accumulation of UPR proteins was independent of transcriptional regulation, or that upregulation of UPR genes occurred in an early and transient fashion that prevented their detection at 6 DPI.

### Ascorbic acid reduces H5‐induced defences and stress symptoms

**Figure 11:**
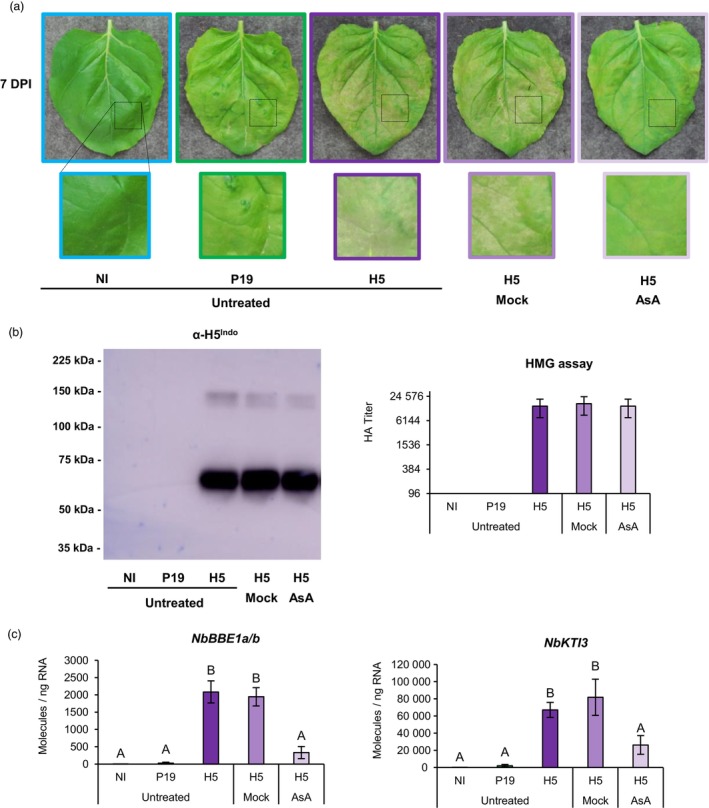
Ascorbic acid reduces H5‐induced defences and stress symptoms. (a) Stress symptoms observed on representative leaves from each condition harvested at 7 DPI. Underneath each leaf picture, a magnified leaf section highlights differences in intensity of the observed symptoms. (b) Western blot depicting H5 protein accumulation (left panel). Hemagglutination (HMG) assay also highlights HA protein activity (right panel). (c) Expression of H5‐induced genes *NbBBE1a/b* and *NbKTI3* as measured by RTqPCR at 7 DPI. Results are expressed in numbers of molecules per ng of RNA. Groups that do not share the same letter are statistically different. Condition names are as follows: NI: non‐infiltrated leaves; P19: leaves infiltrated with AGL1 and expressing P19 only; H5: leaves infiltrated with AGL1 and co‐expressing P19 and H5; H5 Mock: H5‐expressing leaves sprayed at 48 h intervals with a Mock solution; H5 AsA: H5‐expressing leaves sprayed at 48 h intervals with a 10 mm solution of ascorbic acid (AsA).

Several lines of evidence suggested that *Agrobacterium*‐mediated expression of influenza protein H5 results in oxidative stress activation, especially in the apoplast where VLPs have been shown to accumulate (D'Aoust *et al*., 2008). We hypothesized that this response was responsible for necrotic symptoms observed on H5‐expressing leaves (Figure 1a and Figure S2A). To test this, infiltrated plants co‐expressing P19 and H5 were sprayed with a 10 mm solution of AsA. In *N. benthamiana*, sodium ascorbate was previously shown to suppress necrosis induced by expression of human proteins (Nosaki *et al*., 2021). To prevent interference with AGL1 infection and transfer of T‐DNA, the first spray was applied at 2 DPI. Recall treatments were then performed at 48 h intervals, before harvesting at 7 DPI. At harvest, no symptom was observed on NI leaves, while leaves only expressing P19 showed chlorosis (Figure 11a). For leaves co‐expressing P19 and H5, cell death symptoms of similar intensities were observed for untreated plants, or plants sprayed with a Mock solution. For H5‐expressing leaves sprayed with AsA, yellowish discoloration was observed, but little to no necrotic symptoms were denoted. Exogenous application of the antioxidant thus reduced stress symptoms otherwise associated to H5 protein expression.

To confirm that decreased stress symptoms were not simply caused by lower accumulation of the recombinant protein, H5 accumulation and HA activity were monitored by western blotting and HMG assays, respectively. Results showed similar H5 protein levels and similar HA activity in untreated, Mock‐treated or AsA‐treated leaves expressing H5 (Figure 11b). This confirmed that AsA improved biomass quality at harvest without interfering with H5 protein expression or activity. To assess whether AsA also reduced defence signalling associated with H5 protein expression, RTqPCR was performed using genes previously characterized as good markers of H5‐induced oxidative stress (*NbBBE1a/b*; Figure 5c), or oxylipin response (*NbKTI3*; Figure 8a). In both cases, results showed barely detectable transcript levels in NI and P19 samples (Figure 11c), confirming H5‐specific regulation of these genes. For untreated and Mock‐treated leaves expressing H5, defence genes were similarly induced. Gene upregulation was also detected in H5 leaves sprayed with AsA, however expression levels were significantly lower than those detected in untreated and Mock‐treated leaves expressing H5, in addition to being not statistically different from those seen in NI and P19 samples (Figure 11c). Reduced expression of the defence genes was thus consistent with intensity of the necrotic symptoms observed on H5‐expressing leaves (Figure 11a).

## Discussion

**Figure 12:**
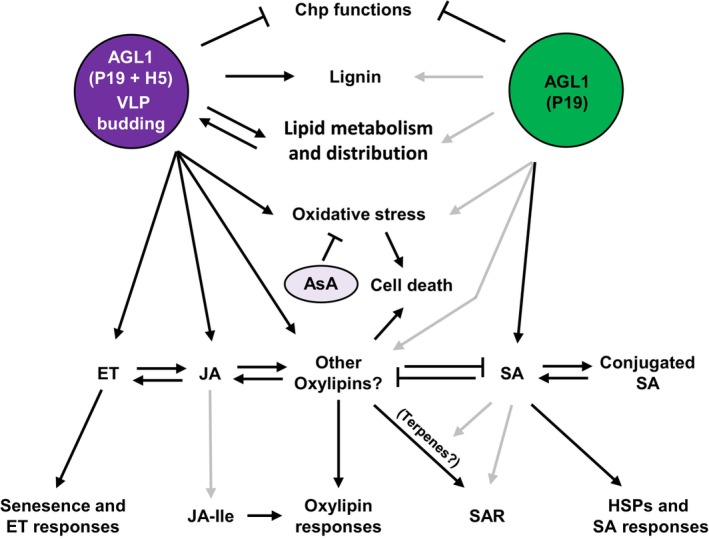
Model of plant responses to P19 and VLP expression. In response to foreign protein expression, some plant responses are shared between conditions, while others are specific to either expression of P19 only (green) or co‐expression of P19 and H5 proteins (purple; see text for details). Black and grey arrows indicate strong or weak activation, respectively. AsA, ascorbic acid; Chp, chloroplast; ET, ethylene; HA, hemagglutinin; HSP, heat shock protein; JA, jasmonic acid; JA‐Ile, jasmonic acid isoleucine conjugate; SA, salicylic acid; SAR, systemic acquired resistance; VLP, virus‐like particle.

In characterizing the effects of *Agrobacterium*‐mediated expression of foreign proteins in *N. benthamiana*, we found a strong convergence in the datasets collected through RNAseq, RTqPCR and proteomics. These gene and protein expression patterns were also consistent with quantification of defence metabolites, including lignin and key stress hormones. Taken as a whole, these results thus provide a comprehensive overview of the complex interplay of responses taking place following expression of P19, or co‐expression of P19 and H5 that leads to the formation of VLPs (Figure 12). Transient expression of these foreign proteins pointed to a trade‐off between plant growth and immunity, as exemplified by the down‐regulation of CRGs and concomitant activation of several defence pathways. Among the latter, some were co‐induced by AGL1 infiltration, expression of P19 only, or co‐expression of P19 and H5 proteins. Generally, co‐induced pathways were however affected at much higher levels when the H5 protein was expressed. On the other hand, some defence responses appeared to be more specific to a defined condition, including enhanced expression of HSPs in P19 samples, or induction of lipid‐ and oxylipin‐related responses in H5 samples (Figure 12).

Induced expression of genes encoding cytosolic HSPs is common during heat shock stress, which is known to interfere with general protein folding. Upregulation of these genes in P19 samples was thus consistent with higher accumulation of the VSR (Figure 1c), and with its sub‐cellular localization in the cytosol. Upregulation of *HSP* genes is also a well‐known mark of SA signalling (Sangwan *et al*., 2022; Snyman and Cronjé, 2008), consistent with enhanced SA accumulation and induction of other SA response genes in P19 samples (Figure 7). Interestingly, no expression of *HSP* genes was seen in H5 samples (Figure 3), despite confirmed accumulation of the P19 protein in this condition (Figure 1c). Keeping in mind that P19 protein level was lower in H5 samples compared to P19 samples, it is yet tempting to speculate that complete dampening of *HSP* gene induction was associated with signalling crosstalk between the SA pathway induced by P19 and the oxylipin pathway induced by H5, similar to how H5 reduced SA accumulation (Figure 7a) and expression of other SA response genes (Figure 7c). In H5 samples, dampening of the SA pathway was further evidenced by higher accumulation of conjugated SA compared to AGL1 or even P19 samples (Figure 7a), despite lower accumulation of the VSR when co‐expressed with H5 (Figure 1c).

Identification of H5‐specific responses indicated that the secreted protein, or the accumulation of its associated VLPs, results in a unique molecular signature that deeply reshapes the metabolism of plant cells. Among H5‐induced genes, some encoded proteins that are involved in PA and LPA accumulation, or in the control of lipid distribution within membranes (Table S4; Figure 4). Accumulation of PA likely contributes to plant immunity, as this metabolite is a well‐known secondary messenger involved in stress signalling (Lim *et al*., 2017). Alternatively, accumulation of specific lipids and their altered distribution within membranes may be consequences of H5 protein expression on the endomembrane system, including the production and budding of VLPs (Figure 12). Indeed, PA, DAG and LPA exhibit conical shapes, and their localized accumulation promotes membrane bending rather than formation of planar bilayers (Roth, 2008). Enhanced accumulation and/or altered distribution of these lipids within membranes thus constitute key mechanisms to modulate membrane curvature. Interestingly, localized accumulation of conical‐shaped lipids within the PM is exploited by the influenza virus during mammalian host cell entry and exiting via membrane budding (Oguin *et al*., 2014). As for true influenza virions, we thus hypothesize that modifications in lipid composition and distribution within membranes were due to feedback mechanisms caused by high production of the secreted H5 protein and budding of its associated VLPs.

Transcriptomics also revealed that wounding and herbivory response genes were highly induced in H5‐expressing samples (Figure 12). Quantification of jasmonates accordingly showed H5‐specific accumulation of JA, but interestingly not of JA‐Ile (Figure 8b). In P19 and H5 samples, genes promoting accumulation of JA‐Ile were correspondingly repressed (Figure 8e), suggesting that H5 expression results in oxylipin‐related responses that do not uniquely (or mainly) proceed through the JA pathway and that other bioactive oxylipins are produced in this condition (Figure 12). While these signalling compounds remain to be formally identified, oxidized polyunsaturated fatty acids, divinyl ethers and cyclopentenones such as OPDA are likely candidates. In line with this, oxylipin regulatory genes from alternative branches of the octadecanoid pathway were strongly induced by H5 expression (Figure 9), including 9*‐LOX, α‐DOX* and *CYP74* genes. In tobacco, agroinfiltration was previously shown to induce 9‐LOX activity and corresponding upregulation of *9‐LOX* genes (Huang *et al*., 2010). Likewise, our data shows that several oxylipin regulatory genes were induced in AGL1 and P19 samples, including *9‐LOX* gene *NbLOX1* (Figures 9b,c). Following agroinfiltration, upregulation of these genes was however greatly enhanced by H5 expression (Figure 12). In the same report (Huang *et al*., 2010), 13‐LOX and hydroperoxide lyase (HPL) enzymes were shown to stimulate production of green leaf volatiles (GLVs) such as hexanal (Figure 9a). While 13‐LOX encoding gene *NbLOX3a* was induced in H5 samples, *HPL* gene Niben101Scf00313g08016 (*NbHPL*) was strongly repressed in P19 and H5 samples (Figure 9b; Tables S1 and S9). This suggests that 13‐LOX activity supports JA synthesis in H5 samples (Figure 8b), but that GLV production is not a major component of the plant response to VLPs.

In Arabidopsis and tomato, OPDA has signalling functions independent of JA or JA‐Ile (Bosch *et al*., 2014; Stintzi *et al*., 2001). Genes specifically induced by OPDA have also been identified in Arabidopsis (Ribot *et al*., 2008; Taki *et al*., 2005). In H5 samples, strong and specific upregulation of homologues from these OPDA‐specific genes suggests that this signalling cascade is indeed active during VLP expression. These include *BBE* genes (Figure 5c), phosphate transporter gene Niben101Scf05712g02003 (*NbPHO1;H10*), zinc finger protein (ZFP) genes Niben101Scf05373g01004 (*NbZFP1*) and Niben101Scf10015g02003 (*NbZFP2*), as well as ribonuclease genes Niben101Scf04082g01004 (*NbRNS1*) and Niben101Scf09597g01002 (*NbRNS2*; Tables S1 and S9). For *ZFP* and ribonuclease genes, RTqPCR confirmed strong and H5‐specific induction (Figure S3), further suggesting that oxylipins other than JA and JA‐Ile are actively signalling in this condition (Figure 12).

While partially compromised in SA‐related responses (Figures 3 and 7), H5 samples still displayed induction of SAR‐related genes, including homologues of the well‐known SAR regulatory gene *AZI1* (Figure 7h). As SAR is often linked to SA signalling, this result at first seemed contradictory. JA and other oxylipins have however been shown to induce both local and systemic defence responses (Bosch *et al*., 2014; Vicente *et al*., 2012), perhaps explaining the SAR signature observed in H5‐expressing samples. Alternatively, a subset of SAR‐related genes from Arabidopsis was shown to not only respond to SA, but also to volatile terpene metabolites that promote inter‐plant communication in parallel of SA (Riedlmeier *et al*., 2017). Within that SAR‐related gene subset, *AZI1*, *CHI*, *MLO6* and *BBE* genes can be found, a signature that closely resembles SAR‐related genes induced by H5 expression (Figure 7). To further support this hypothesis, expression of genes involved in terpene synthesis was examined following foreign protein expression. RNAseq revealed upregulation of several genes from the mevalonate pathway, which promotes assembly of terpene backbone molecules (Figure S4A). Also induced, were several genes encoding cytochrome P450 of family 71 (CYP71), which mediate diversification of terpene molecules, so complex terpene blends with refined biological functions can be produced (Banerjee and Hamberger, 2018). While induced expression of some terpene‐related genes was seen in AGL1 and P19 samples, expression was either higher or specific to H5 samples (Figure S4B and Tables S1 and S12). As RTqPCR confirmed upregulation patterns from several of these terpene‐related genes (Figure S4C), results suggest that terpene signalling is another key feature of the present expression system (Figure 12), with expression of H5 leading to stronger and broader activation of this pathway. In turn, stronger and broader terpene signalling in H5 samples may promote SAR despite compromised SA responses in this condition. In a molecular farming context, where agroinfiltrated plants are packed in closed expression chambers, plant‐to‐plant communication would be expected to play a role, especially since the expression phase lasts for several days. Whether these responses are due to intra‐ or inter‐plant signalling however remains to be determined, keeping in mind that leaves from the whole plant shoots were vacuum‐infiltrated (see the Materials and methods section).

Overall, our results highlight diversity and interconnectivity of the responses induced in *N. benthamiana* leaves expressing foreign proteins. While some of these responses will likely vary according to the foreign proteins expressed, data reported here is still of practical significance considering that *Agrobacterium*‐mediated expression is widely employed for molecular farming. Obviously, this unique pathosystem remains artificial, yet it now contributes to solving major public health issues, including worldwide spreading of infectious diseases such as influenza. Despite multiple stresses imposed by the system, plants show remarkable resilience that allows them to produce high amounts of recombinant proteins. Hopefully, our analyses will help to improve molecular farming techniques, as exemplified by the reduction of H5‐induced defences and stress symptoms by AsA (Figures 11 and 12). Although such treatments would be challenging to implement for commercial‐scale production of VLPs, these results nonetheless demonstrate the importance of plant immunity during foreign protein expression. They also confirm that hypothesis‐driven strategies can be applied to improve productivity, or as in this case biomass quality. Based on the knowledge developed here, alternative improvement strategies can also be envisioned, including targeted editing of the host plant genome and heterologous co‐expression of helper proteins whose activities (protein folding, cell detoxification, signalling, etc.) can lead to a cellular environment more favourable to the production of biopharmaceutical products such as VLPs or antibodies (Goulet *et al*., 2012; Grosse‐Holz *et al*., 2018; Jutras *et al*., 2015).

## Materials and methods

### Seed germination and plant growth

Seeds of *N. benthamiana* were spread on pre‐wetted peat mix plugs (Ellepot) and placed in a germination chamber for 2 days, where conditions were as follows: 28 °C/28 °C day/night temperature, 16 h photoperiod, 90% relative humidity and light intensity of 7 μmol/m^2^/s. Germinated plantlets were next transferred in a growth chamber for 15 days, where conditions were as follows: mean temperature of 28 °C over 24 h, 16 h photoperiod, mean relative humidity of 66% over 24 h, 800 ppm carbon dioxide (CO_2_) injected only during the photo‐phase and light intensity of 150 μmol/m^2^/s. During this time, watering and fertilization were provided as needed. After 2 weeks, peat mix plugs were transferred to four inches pots containing pre‐wetted peat‐based soil mix (Agro‐Mix). Freshly transferred plantlets were then moved to a greenhouse, where conditions were as follows: mean temperature of 25 °C over 24 h, 16 h photoperiod, mean relative humidity of 66% over 24 h, 800 to 1000 ppm CO_2_ injected only during the photo‐phase and light intensity according to natural conditions, but supplemented with artificial high pressure sodium lights at 160 μmol/m^2^/s. In the greenhouse, watering and fertilization were provided as needed. Growth was allowed to proceed for an average of 20 additional days, until the plants were ready for agroinfiltration.

### Binary vector constructs

For VLP expression, sequences from the mature HA protein of pandemic influenza virus strain H5 Indonesia (H5/A/Indonesia/05/2005; H5^Indo^) were fused to the signal peptide of a *Medicago sativa* (alfalfa) PDI using PCR‐based methods. Once assembled, chimeric *H5* gene was reamplified by PCR and then introduced in the T‐DNA region of a customized pCAMBIA0380 binary vector previously linearized with restriction enzymes *SacII* and *StuI* using the In‐Fusion cloning system (Clontech). Expression of *H5* was driven by a *2X35S* promoter from the cauliflower mosaic virus (CaMV). The expression cassette also comprised 5′‐ and 3′‐untranslated regions (UTRs) from the cowpea mosaic virus (CPMV), and the *Agrobacterium nopaline synthase* (*NOS*) gene terminator. To prevent silencing induced by recombinant gene expression *in planta*, T‐DNA region of the binary vector used to express *H5* also included the suppressor of RNA silencing gene *P19*, under the control of a *plastocyanin* promoter and terminator. For P19 samples, a binary vector allowing expression of P19 only was employed. For AGL1 samples, a binary vector harbouring a frameshifted version of *P19* was used as a control.

### *Agrobacterium* cultures and plant infiltration

Binary vectors were transformed by heat shock in *Agrobacterium* strain AGL1. Transformed bacteria were plated on Luria‐Bertani (LB) medium, with appropriate antibiotics selection (kanamycin 50 μg/mL). Colonies were allowed to develop at 28 °C for 2 days. Using isolated colonies, frozen glycerol stocks were prepared and placed at −80 °C for long‐term storage. When ready, frozen bacterial stocks were thawed at room temperature before transfer in pre‐culture shake flasks containing LB medium with antibiotics selection (kanamycin 50 μg/mL). Bacterial pre‐cultures were grown for 18 h at 28 °C with shaking at 200 rpm. While keeping kanamycin selection, pre‐cultures were transferred to larger shake flasks and bacteria were allowed to develop for an extra 18 h at 28 °C with shaking at 200 rpm. Using a spectrophotometer (Implen), bacterial inoculums were prepared by diluting appropriate volumes of the bacterial cultures in resuspension buffer (10 mm MgCl_2_, 5 mm MES, pH 5.6). For AGL1, P19 and H5 samples, a final OD_600_ of 0.6 was employed for all experiments. Vacuum infiltration was performed by placing whole plant shoots upside down in an airtight stainless‐steel tank containing the appropriate bacterial suspension. To draw air out of the leaves, vacuum pressure was applied for 1 min before pressure release to force the bacterial inoculum into the leaves.

### Transient protein expression and biomass harvesting

Recombinant protein accumulation was allowed to proceed for 6 or 7 days, as indicated. For all experiments, expression took place in condition‐controlled plant growth chambers, where settings were as follows: 20 °C/20 °C day/night temperature, 16 h photoperiod, 80% relative humidity and light intensity of 150 μmol/m^2^/s. Watering was performed every other day, with no fertilizer supplied during the expression phase. For biomass harvesting, leaves of similar developmental stage were selected using the leaf plastochron index (Meicenheimer, 2014). The fourth and fifth fully expanded leaves starting from the top of each plant were harvested without petiole. Freshly cut leaves were placed in pre‐frozen 50 mL Falcon tubes, before flash freezing in liquid nitrogen. Frozen biomass was stored at −80 °C until ready for analysis. Using pre‐chilled mortars and pestles, foliar tissue was ground and homogenized into powder using liquid nitrogen. Each sample was made from four leaves collected on two randomly selected plants. The average results presented were obtained from at least three biological replicates.

### Protein extraction, western blotting and HMG assays

For protein extraction, 1 g of frozen biomass powder was taken out of the −80 °C freezer and placed on ice. A 2 mL volume of extraction buffer (50 mm Tris, 500 mm NaCl, pH 8.0) was added, followed by 20 μL of 100 mm phenylmethanesulfonyl fluoride (PMSF) and 2 μL of 0.4 g/mL metabisulfite. Quickly after addition of all solutions, samples were crushed for 45 s using a Polytron homogenizer (ULTRA‐TURRAX® T25 basic) at maximum speed. One mL of each sample was then transferred to a pre‐chilled Eppendorf tube and centrifuged at 10 000 ***g*** for 10 min at 4 °C. Supernatants were carefully recovered, transferred to new Eppendorf tubes and kept on ice until determination of protein concentration. To quantify protein content from crude extracts, the Bradford method was employed, with bovine serum albumin as a protein standard.

For western blotting, total protein extracts were diluted in extraction buffer and mixed with 5X Laemmli sample loading buffer to reach a final concentration of 0.5 μg/μL. Protein samples were denatured at 95 °C for 5 min, followed by a quick spin using a microcentrifuge. 20 μL of each denatured protein extract (10 μg) was then loaded on Criterion™ XT Precast polyacrylamide gels 4%–12% Bis‐Tris and separated at 110 volts for 105 min. Using transfer buffer (25 mm Tris, 192 mm Glycine, 10% methanol), proteins were next electro transferred onto a polyvinylidene difluoride (PVDF) membrane at 100 volts. After 30 min, membranes were placed in blocking solution: 1X Tris‐Buffered Saline with Tween‐20 (TBS‐T; 50 mm Tris, pH 7.5, 150 mm NaCl, 0.1% (v/v) Tween‐20), with 5% nonfat dried milk. Membranes were blocked overnight at 4 °C with gentle shaking. The next morning, blocking solution was removed and primary antibodies were incubated at room temperature for 60 min with gentle shaking in 1X TBS‐T, 2% nonfat dried milk solution. After four washes in 1X TBS‐T, secondary antibodies were added and incubated at room temperature for 60 min with gentle shaking in 1X TBS‐T, 2% nonfat dried milk solution. After four extra washes in 1X TBS‐T, Luminata™ Western HRP Chemiluminescence Substrate (Thermo Fisher Scientific) was added to the membranes and protein complexes were visualized under the chemiluminescence mode of an Imager 600 apparatus (Amersham). Antibody dilutions were as follows: anti‐HA A/Indonesia/05/2005 (H5N1; CBER): 1/5000 (primary antibody). Rabbit anti‐sheep (JIR): 1/10 000 (secondary antibody).

For HMG assays, turkey red blood cells were diluted to a concentration of 0.25% (v/v) in phosphate‐buffered saline solution (PBS; 0.1 M PO_4_, 0.15 M NaCl, pH 7.2). While keeping red blood cells on ice, protein samples were diluted in extraction buffer using 1/384 and 1/576 ratios. For each dilution, 200 μL of total protein extract was transferred to the first row of a 96‐well plate. Eight serial dilutions were then performed using 100 μL of protein extract mixed to 100 μL of PBS buffer previously poured in each plate well. Following serial dilutions, 100 μL of the red blood cell solution was added to protein extracts. After thorough mixing, samples were incubated overnight at room temperature. HA activity was scored visually on the next day.

### RNA extractions and quantification

Using the RNeasy commercial kit (Qiagen), 100 mg of frozen biomass powder was used for RNA extractions. Residual DNA was removed using the RNase‐free DNase Set (Qiagen). Concentration of RNA extracts was determined using a spectrophotometer (Implen) and integrity evaluated using a 2100 BioAnalyzer (Agilent). For long‐term storage, RNA extracts were stabilized by adding the RNAseOUT recombinant ribonuclease inhibitor (Thermo Fisher Scientific), before freezing at −80 °C until ready for further analysis.

### RTqPCR analyses

For each sample, 1 μg of RNA was reverse transcribed into cDNA using the QuantiTect Reverse Transcription Kit (Qiagen). Transcript quantification was performed in 96‐well plates, using the ABI PRISM 7500 Fast real‐time PCR system and custom data analysis software (Thermo Fisher Scientific). Each reaction contained the equivalent of 5 ng cDNA as a template, 0.5 μm of forward and reverse primers, and 1X QuantiTect SYBR Green Master Mix (Qiagen) for a total reaction volume of 10 μL. RTqPCR runs were done under the SYBR Green amplification mode and cycling conditions were as follows: 15 min incubation at 95 °C, followed by 40 amplification cycles at 95 °C for 5 s, 60 °C for 30 s and 65 °C for 90 s. Reactions in the absence of cDNA template were conducted as negative controls and melting curve analyses were performed to confirm lack of primer dimer formation and amplification specificity. Resulting fluorescence and cycle threshold (Ct) values were next exported to the Microsoft Excel software. To correct for biological variability and technical variations during RNA extraction, RNA quantification or reverse transcription, expression from six housekeeping genes (*NbACT1*, *NbVATP*, *NbSAND*, *NbUBQ1*, *NbEF1‐α* and *NbGAPDH1*) was used to normalize expression data (Vandesompele *et al*., 2002). Stable expression of housekeeping genes was verified by RTqPCR (Figure S5) and using RNAseq data (Table S13). Normalized numbers of molecules per ng of RNA were deduced using the 2^−ΔΔCt^ method (Bustin *et al*., 2009; Livak and Schmittgen, 2001) and standard curves derived from known quantities of phage lambda DNA. Standard deviation related to the within‐treatment biological variation was calculated in accordance with the error propagation rules. Sequences of all primers used in this study are available in the Table S14.

### RNAseq analyses

To study global changes to the plant transcriptome, RNA extracts were placed on dried ice and shipped to an external service provider (Génome Québec; McGill University, Montréal). Briefly, ribosomal RNA (rRNA) was depleted to concentrate messenger RNA (mRNA). After fragmentation, mRNA stranded nucleotide libraries were created by fusing TruSeq RNA sequencing adapters (Illumina), followed by library amplification by PCR. High throughput sequencing was performed using a HiSeq 2000 SR100 device (Illumina). Three biological replicates from each experimental condition were sequenced. For each library, an average of 36 954 631 reads were obtained and a total of 3 695 463 144 bases were sequenced. Following filtering and quality control steps, sequences were aligned against the nuclear genome of *N. benthamiana* (https://solgenomics.net/). Once aligned and quantified, sequencing data was used to perform pairwise comparisons between conditions, as indicated.

### Large‐scale proteomics

To study changes in protein abundance, total protein extracts from three biological replicates of each condition were used for iTRAQ labelling. Detailed description of sample preparation, peptide labelling and mass spectrometry methods are available elsewhere (Jutras *et al*., 2020). Peptide mass spectra were analysed using the Mascot database search engine (Matrix Science), which is implemented in Proteome Discoverer (Thermo Scientific). The search algorithm was set to interrogate the Solanaceae protein database from UniProt, which comprises over 117 800 proteins (http://www.uniprot.org/taxonomy/4070). Common peptide contaminants like keratin were taken out of the analysis.

### Quantification of stress hormones

For quantification of salicylates and jasmonates, 500 mg of fresh leaf tissue was employed. For quantification of ACC, 1 g of frozen leaf biomass powder was lyophilized for 48 h to obtain 50 mg of dried tissue. Once weighted, samples were placed on dry ice and shipped to Dr. Irina Zaharia at the National Research Council of Canada located in Saskatoon, Saskatchewan, Canada. Hormone quantification was performed using high‐performance liquid chromatography–electrospray ionization–tandem mass spectrometry (HPLC‐ESI‐MS/MS), using the ACQUITY UPLC system equipped with a binary solvent delivery manager and a sample manager (Waters). This system was also coupled to a Micromass Quattro Premier XE quadrupole tandem mass spectrometer (Waters). MassLynx and QuanLynx softwares (Micromass) were used for data acquisition and analysis. Quantification data was obtained from comparison with standard‐derived calibration curves.

### Lignin quantification

Lignin quantification was performed using an acid‐catalysed reaction that forms soluble lignin‐thioglycolate complexes. Suitable for photometric measurements, this method was developed in spruce (*Picea abies*; Lange *et al*., 1995), but later adapted for use in *N. benthamiana* (de la Torre *et al*., 2014). For each sample, 200 mg of grounded biomass was used. Quantification of lignin was obtained by measuring absorbance at 280 nm and comparison with standard curves made using known quantities of purified lignin polymers (Sigma).

### Ascorbic acid treatments

Purified AsA was purchased from Sigma. A 10 mm solution was obtained by dilution in 20 mm MES (pH 6.0), supplemented with 0.05% Tween 20. After solubilization of AsA, the pH was readjusted to 6.0 using 5 N NaOH, before spraying on the plants. For each treatment, blocks of 12 plants were uniformly sprayed with a 50 mL volume of the AsA solution, or with a similar volume of the mock solution at pH 6.0. A first treatment was applied at 2 DPI. Recall treatments were then performed at 4 and 6 DPI, before harvesting at 7 DPI. Untreated NI, P19 and H5 plants were used as controls.

### Statistical analyses

Statistical analyses were performed on Graph Pad Prism 9.5.0. Groups were analysed using one‐way ANOVA followed by a post hoc Tukey's multiple comparison test with an alpha threshold of 0.05. Groups are labelled with a compact letter display. Groups not sharing the same letter are statistically different.

### Accession numbers

Raw sequencing data from the RNAseq study is available on the Gene Expression Omnibus (GEO) website under the accession number GSE233178.

## Funding

Funding for this work was provided by Medicago Inc. This work was also supported by Collaborative Research and Development grants from the Natural Sciences and Engineering Research Council of Canada to Dominique Michaud (CRDPJ 495852) and Peter Moffett (CRDPJ 477619).

## Conflict of interest

At the time of this work, L.P.H., R.T., F.P.G., M.E.P., E.R., M.A.C., P.O.L and M.A.D. were employees of Medicago Inc. Other authors declare that the research was conducted in the absence of any commercial or financial relationships that could be construed as a potential conflict of interest.

## Author contributions

L.P.H., D.M., P.M. and M.A.D. designed the research, supervised the project and analysed the data. L.P.H. and M.A.C. drafted the manuscript and assembled the figures. P.O.L. managed production of genetic constructs. R.T. performed RTqPCR, HMG assays and western blots. F.P.G. and M.A.C. performed statistical analyses. L.P.H., A.R. G.G and C.B. performed RNAseq. J.F.L., M.A.C. and F.P.G. generated RNAseq *in silico* data. M.C.G., A.B. and L.P.H. conducted proteomics studies and produced biomass for plant hormone quantification. M.C.G and F.P.G. generated proteomics *in silico* data. M.E.P. performed VLP purification and TEM imaging. E.R. performed lignin quantification. R.T. and L.P.H. performed experiments with AsA. All authors read, helped to edit and approved final version of the manuscript.

## Supporting information


**Figure S1** Phylogenetic relationships between PR1 homologues of *Nicotiana benthamiana*.
**Figure S2** Stress symptoms and analysis of biomass used for proteomics.
**Figure S3** Expression of OPDA‐specific genes.
**Figure S4** Expression of terpene‐related genes.
**Figure S5** Expression of reference genes.

**Table S1** Unsorted lists of genes with significantly altered expression in AGL1, P19 and H5 samples.

**Table S2** Lists of chloroplast‐related genes found in P19 and H5 samples.

**Table S3** Lists of *HSP* and chaperone genes found in P19 and H5 samples.

**Table S4** List of lipid‐related genes found in P19 and H5 samples.

**Table S5** List of genes related to oxidative stress activation found in P19 and H5 samples.

**Table S6** List of genes related to sugar metabolism found in P19 and H5 samples.

**Table S7** List of lignin‐related genes found in P19 and H5 samples.

**Table S8** List of genes related to SA signalling and SAR found in P19 and H5 samples.

**Table S9** Lists of oxylipin‐related genes found in P19 and H5 samples.

**Table S10** List of ET‐ and senescence‐related genes found in P19 and H5 samples.

**Table S11** Unsorted lists of proteins with significantly altered accumulation levels in AGL1, P19 and H5 samples.

**Table S12** List of terpene‐related genes found in P19 and H5 samples.

**Table S13** RNAseq data from reference genes used to normalize RTqPCR results.

**Table S14** List of RTqPCR primers used in this study.

**Appendix S1** List of abbreviations. Definition of all acronyms used in this study.

## Data Availability

All data discussed in this study can be found in the manuscript and in the Supplementary Materials.
