# Supplementary material for: Molecular responses of agroinfiltrated Nicotiana benthamiana leaves expressing suppressor of silencing P19 and influenza virus‐like particles
Source: Plant Biotechnol J. 2023 Dec 2;22(5):1078–100. doi: 10.1111/pbi.14247 (PMC11022802; doi:10.1111/pbi.14247)
Supplement: Supplementary file 1 — Figure S1 Phylogenetic relationships between PR1 homologues of Nicotiana benthamiana. Figure S2 Stress symptoms and analysis of biomass used for proteomics. Figure S3 Expression of OPDA‐specific genes. Figure S4 Expression of terpene‐related genes. Figure S5 Expression of reference genes. [file PBI-22-1078-s013.pdf]

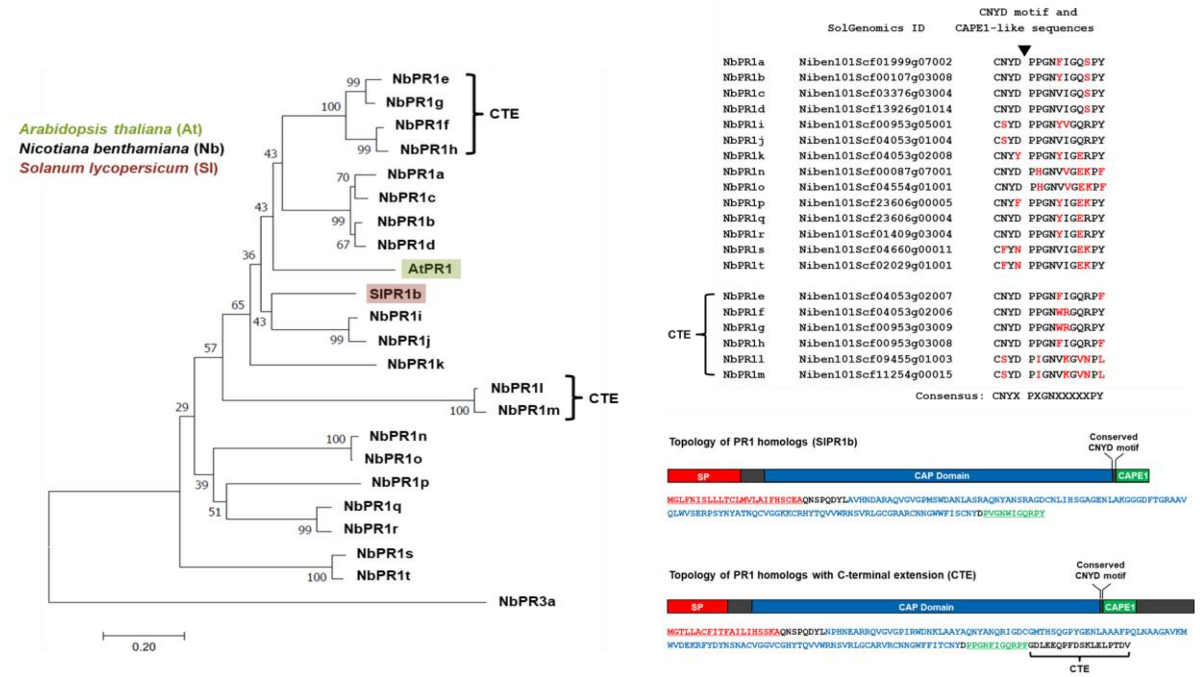

Figure S1. Phylogenetic relationships between PR1 homologs of *N. benthamiana*. The genome of *N. benthamiana* was searched using full-length amino acid sequences of *Solanum lycopersicum* (tomato) SlPR1b (accession number NP_001234314) or Arabidopsis AtPR1 (At2g14610) as queries. Full-length protein sequences that were retrieved were next aligned with ClustalW, using chitinase NbPR3a (Niben101Scf07491g00003) as an outgroup. Alignment parameters were as follow: for pairwise alignment, gap opening, 10.0, and gap extension, 0.1; for multiple alignment, gap opening, 10.0, and gap extension, 0.20. Resulting alignments were submitted to the MEGA5 software and a neighbor-joining tree derived from 5000 replicates was generated. Bootstrap values are indicated on the node of each branch. At the top right, gene model number from corresponding *NbPR1* genes are shown, along with amino acid sequences of the conserved CNYD motif and CAPE1 peptide. A black arrow indicates clipping site of the CAPE1 peptide. At the bottom right, topologies from PR1 proteins with or without a C-terminal extension (CTE) are shown. Abbreviations: At, *Arabidopsis thaliana*; Nb, *Nicotiana benthamiana*; Sl, *Solanum lycopersicum*; SP, signal peptide.

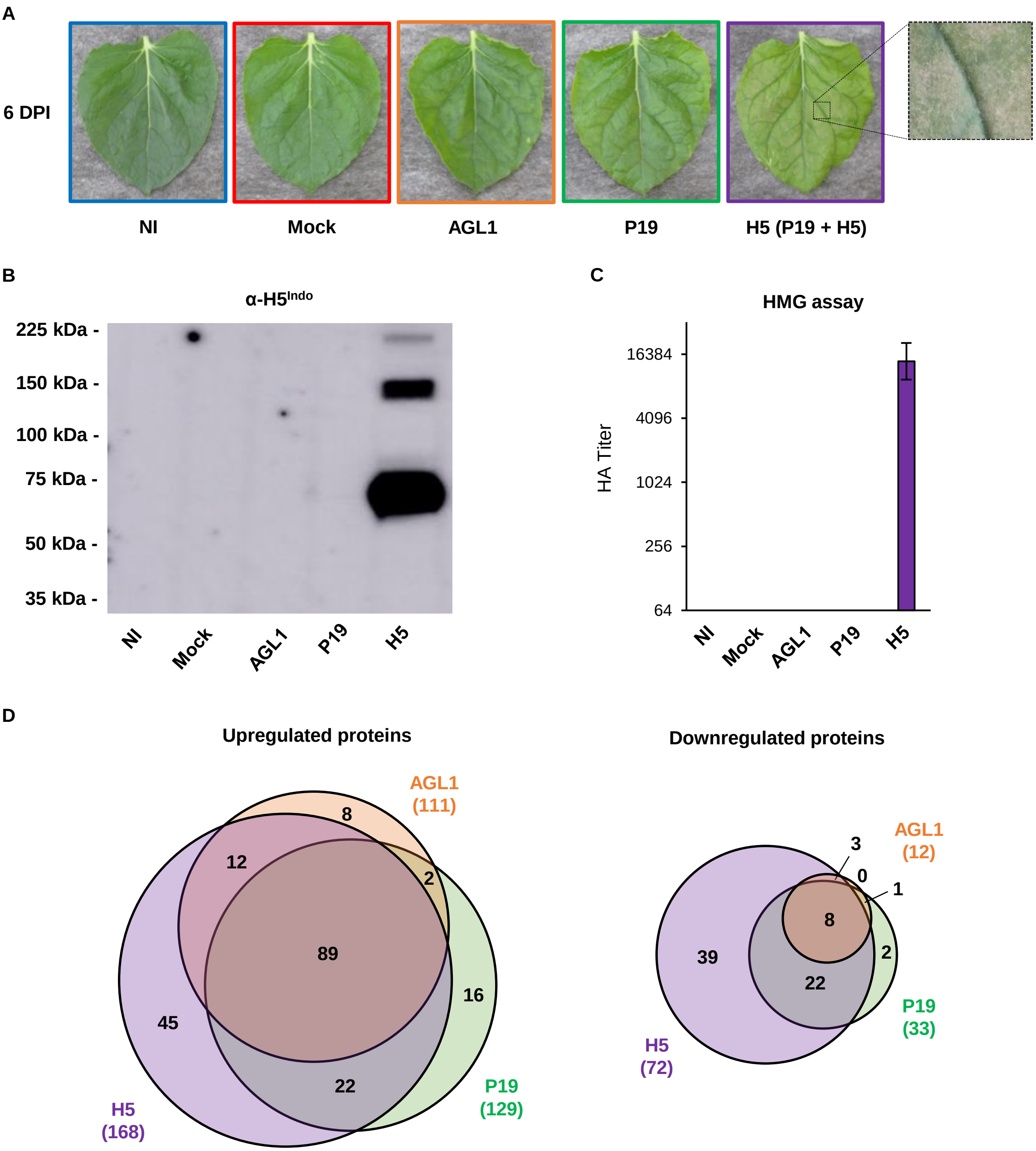

Figure S2. Stress symptoms and analysis of biomass used for proteomics. (A) Stress symptoms observed on representative leaves from each condition harvested at 6 DPI. A magnified leaf section highlights necrotic flecking seen when expressing H5. (B) Western blot confirming H5 protein accumulation. (C) Hemagglutination (HMG) assay confirming HA protein activity. (D) Venn diagrams depicting up- and downregulated proteins after pairwise comparisons: AGL1 *vs* Mock, P19 *vs* Mock, and H5 *vs* Mock. Circle size is proportional to the number of significantly regulated proteins. Proteins specific to AGL1 samples are shown in orange. Proteins specific to P19 samples are shown in green. Proteins specific to H5 samples are shown in purple. Diagram intersects show proteins common to more than one condition. Condition names are as follows: NI: non-infiltrated leaves; Mock: leaves infiltrated with buffer only; AGL1: leaves infiltrated with *Agrobacterium* strain AGL1 that carry a binary vector control; P19: leaves infiltrated with AGL1 and expressing P19 only; H5: leaves infiltrated with AGL1 and co-expressing P19 and H5.

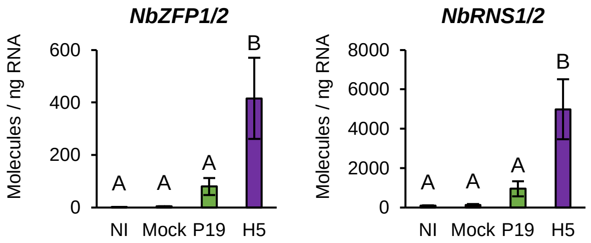

Figure S3. Expression of OPDA-specific genes. Expression of genes specifically induced by OPDA as measured by RTqPCR at 6 DPI. Results are expressed in numbers of molecules per ng of RNA. Groups that do not share the same letter are statistically different. Condition names are as follows: NI: non-infiltrated leaves; Mock: leaves infiltrated with buffer only; P19: leaves infiltrated with AGL1 and expressing P19 only; H5: leaves infiltrated with AGL1 and co-expressing P19 and H5.

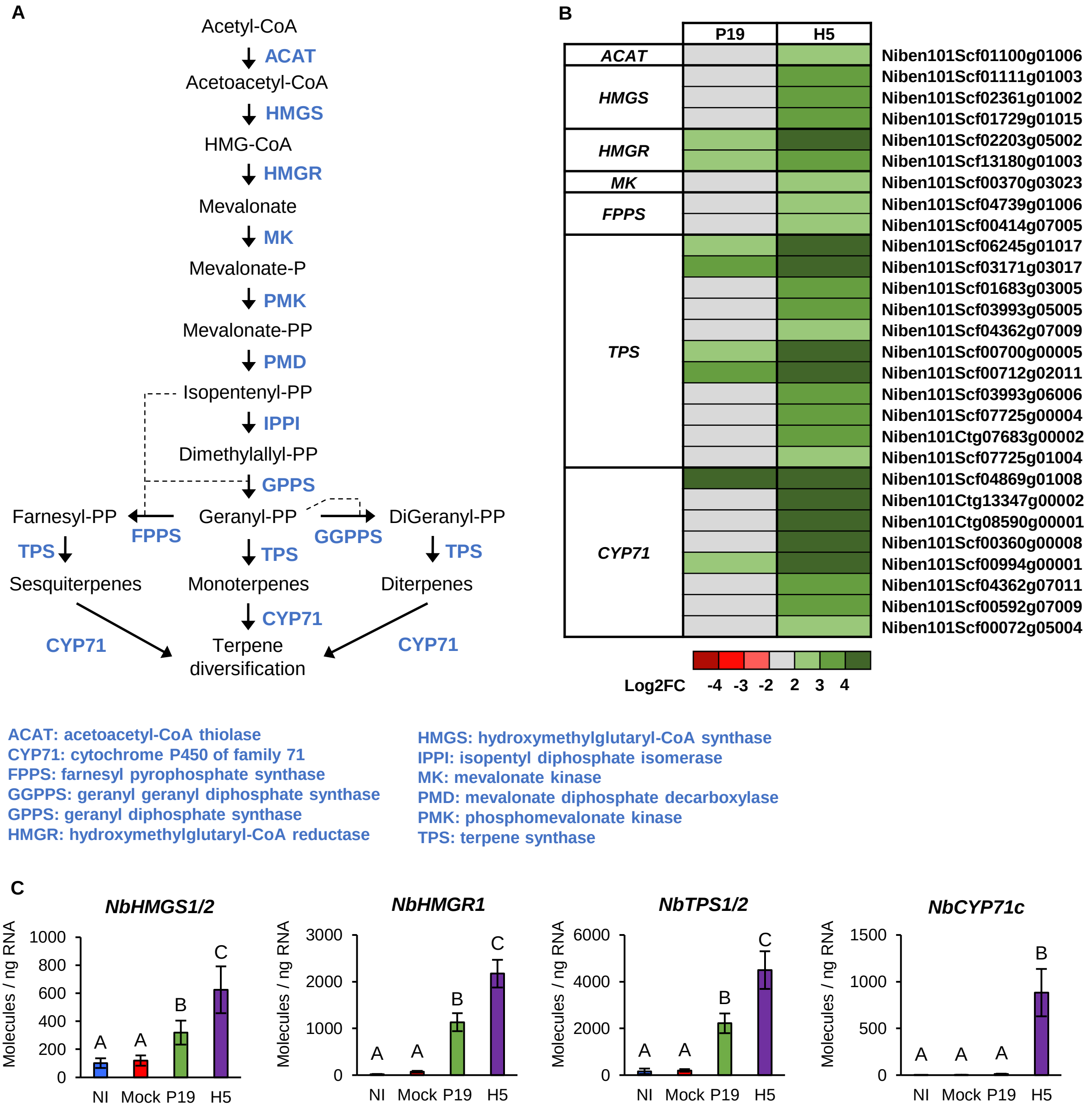

Figure S4. Expression of terpene-related genes. (A) Overview of the mevalonate pathway. Metabolic intermediates are shown in black while enzymes are shown in blue. (B) Heat-map depicting expression of genes involved in terpene synthesis and diversification at 6 DPI. Each line represents a gene shown in the Table S12. Grey indicates genes that are not differentially expressed. Green are red colored gradients respectively reflect extent of gene up- and downregulation, as indicated. (C) Expression of genes involved in terpene synthesis and diversification as measured by RTqPCR at 6 DPI. Results are expressed in numbers of molecules per ng of RNA. Groups that do not share the same letter are statistically different. Condition names are as follows: NI: non-infiltrated leaves; Mock: leaves infiltrated with buffer only; P19: leaves infiltrated with AGL1 and expressing P19 only; H5: leaves infiltrated with AGL1 and co-expressing P19 and H5.

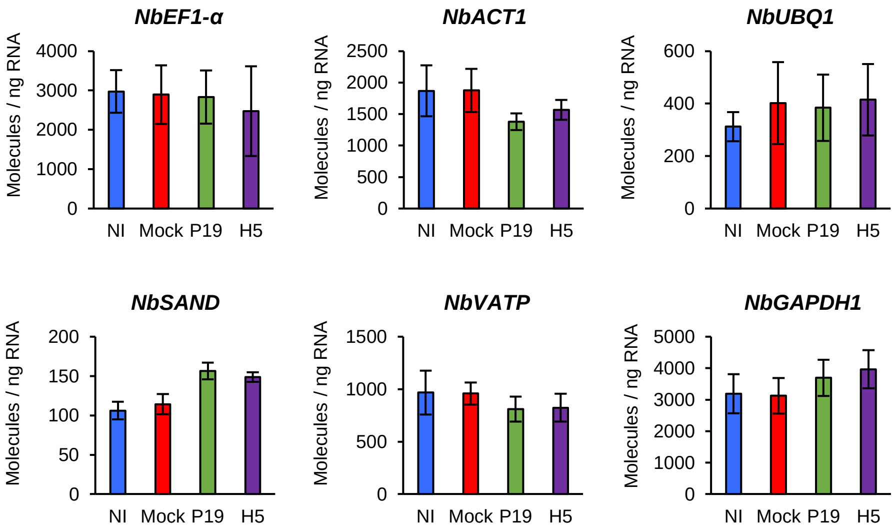

Figure S5. Expression of reference genes. Expression of housekeeping genes used to normalize RTqPCR data at 6 DPI. Results are expressed in numbers of molecules per ng of RNA. No statistically significant difference was observed between the groups. Condition names are as follows: NI: non-infiltrated leaves; Mock: leaves infiltrated with buffer only; P19: leaves infiltrated with AGL1 and expressing P19 only; H5: leaves infiltrated with AGL1 and co-expressing P19 as well as H5.
