# Supplementary material for: Molecular responses of agroinfiltrated Nicotiana benthamiana leaves expressing suppressor of silencing P19 and influenza virus‐like particles
Source: Plant Biotechnol J. 2023 Dec 2;22(5):1078–100. doi: 10.1111/pbi.14247 (PMC11022802; doi:10.1111/pbi.14247)
Supplement: Supplementary file 16 — Appendix S1 List of abbreviations. Definition of all acronyms used in this study. [file PBI-22-1078-s014.docx]

**Supporting information (Hamel *et al.,* 2023)**

**Molecular responses of agroinfiltrated *Nicotiana benthamiana* leaves expressing suppressor of silencing P19 and influenza virus-like particles**

**List of abbreviations.** Definition of all acronyms used in this study.

ACAT Acetoacetyl-CoA thiolase

ACC 1-aminocyclopropane-1-carboxylate

ACO ACC oxidase

ACS ACC synthase

ACT1 Actin 1

ACX Acyl-CoA oxidase

ALD1 AGD2-Like Defense 1

AGL1 *Agrobacterium* strain used in this study

ALA Aminophospholipid ATPase

ALIS ALA-Interacting Subunit

AO Ascorbate oxidase

AOC Allene oxide cyclase

AOS Allene oxide synthase

AsA Ascorbic acid

ASN Asparagine synthetase

AZI1 Azelaic Acid-Induced 1

BBE Berberine-bridge enzyme

BiP Binding immunoglobulin protein

CAD Cinnamyl alcohol dehydrogenase

CaMV Cauliflower mosaic virus

CCoAOMT Caffeoyl-CoA O-methyltransferase

CCR Cinnamoyl-CoA reductase

cDNA complementary DNA

C3H Coumarate-3-hydroxylase

C4H Cinnamate-4-hydroxylase

CHI Chitinase

CHLM Magnesium-protoporphyrin IX methyltransferase

Chp Chloroplast

4CL 4-coumarate-CoA ligase

CO_2_ Carbon dioxide

COMT Caffeic acid 3-O-methyltransferase

CPMV Cowpea mosaic virus

CRG Chloroplast-related gene

CRT Calreticulin

Ct Cycle threshold

CTE C-terminal extension

CWI Cell wall invertase

CYP71 Cytochrome P450 of family 71

CYP74 Cytochrome P450 of family 74

D2 Reaction center protein of the photosystem II

DAG 1,4,5-trisphosphate and diacyl glycerol

DES Divinyl ether synthase

DGK Diacylglycerol kinase

DNA Deoxyribonucleic acid

DOX α-dioxygenase

DPI Days post-infiltration

DW Dry weight

EAS Epoxyalcohol synthase

EF1-α Elongation Factor 1α

EH Epoxide hydrolase

ER Endoplasmic reticulum

ESI Electrospray ionization

ET Ethylene

FC Fold change

F5H Ferulate-5-hydroxylase

FPPS Farnesyl pyrophosphate synthase

FW Fresh weight

GAPDH1 Glyceraldehyde 3-phosphate dehydrogenase 1

GEO Gene Expression Omnibus

GGPPS Geranyl geranyl diphosphate synthase

GLK Golden 2-Like protein

GLV Green leaf volatile

GPAT Glycerol-3-phosphate acyltransferase

GPPS Geranyl diphosphate synthase

HA Hemagglutinin

HCT Hydroxycinnamoyl-CoA transferase

HEMA1 Glutamyl-tRNA reductase involved in chlorophyll synthesis

HIN1 Harpin-induced 1

HMG Hemagglutination

HMGR Hydroxymethylglutaryl-CoA reductase

HMGS Hydroxymethylglutaryl-CoA synthase

HPL Hydroperoxide lyase

HPLC High-performance liquid chromatography

HSP Heat shock protein

ICS Isochorismate synthase

IPPI Isopentyl diphosphate isomerase

iTRAQ Isobaric tags for relative and absolute quantitation

JA Jasmonic acid

JA-Ile Jasmonate-isoleucine conjugate

JAR Jasmonate-Resistant

KTI Kunitz trypsin inhibitor

LAC Laccase

LB Luria-Bertani

LHCB1 Light-harvesting chlorophyll a/b binding protein 1

Log2FC Log2 of the expression fold change value

9-LOX 9-lipoxygenase

13-LOX 13-lipoxygenase

LPA Lysophosphatidic acid

LURP1 Late upregulated in response to *Hyaloperonospora parasitica* 1

MK Mevalonate kinase

MLO6 Mildew Resistance Locus O 6

mRNA Messenger RNA

MS/MS Tandem mass spectrometry

NI Non-infiltrated

NOS Nopaline synthase

OPDA 12-oxophytodienoic acid

OPR OPDA reductase

ORE1 ORESARA 1

PA Phosphatidic acid

padj Adjusted p-value

PAGE Polyacrylamide gel electrophoresis

PAL Phenylalanine ammonia lyase

PAT Patatin

PBS Phosphate-Buffered Saline

PDF Plant defensin

PDI Protein disulfide isomerase

PI Protease inhibitor

PLA Phospholipase A

PLC Phospholipase C

PLD Phospholipase D

PLIP PLASTID LIPASE

PM Plasma membrane

PMD Mevalonate diphosphate decarboxylase

PMK Phosphomevalonate kinase

PMSF Phenylmethanesulfonyl fluoride

PPO Polyphenol oxidase

PR1 Pathogenesis Related 1

PR2 Pathogenesis Related 2

PR3 Pathogenesis Related 3

PR4 Pathogenesis Related 4

PRX Peroxidase

PVDF Polyvinylidene difluoride

RbcL RuBisCO large subunit

RbcS RuBisCO small subunit

RBOH Respiratory burst oxidase homolog

RNA Ribonucleic acid

ROS Reactive oxygen species

rRNA Ribosomal RNA

RuBisCO Ribulose-1,5-bisphosphate carboxylase-oxygenase

RTqPCR Real time quantitative polymerase chain reaction

SA Salicylic acid

SAGT1 SA glucosyl transferase 1

SAR Systemic acquired resistance

SDS Sodium dodecyl sulfate

SGR1 STAYGREEN 1

STP Sugar transport protein

TBS Tris-Buffered Saline

TD Threonine deaminase

TEM Transmission electron microscopy

TF Transcription factor

TPS Terpene synthase

UBQ1 Ubiquitin 1

UPR Unfolded protein response

UTR Untranslated region

VATP Vacuolar ATPase

VLP Virus-like particle

VSR Viral suppressor of RNA silencing

ZFP Zinc finger protein
